# GABP couples oncogene signaling to telomere regulation in TERT promoter mutant cancer

**DOI:** 10.1016/j.celrep.2022.111344

**Published:** 2022-09-20

**Authors:** Andrew M. McKinney, Radhika Mathur, Nicholas O. Stevers, Annette M. Molinaro, Susan M. Chang, Joanna J. Phillips, Joseph F. Costello

**Affiliations:** 1 Department of Neurological Surgery, University of California, San Francisco, San Francisco, CA 94143, USA; 2 Department of Epidemiology and Biostatistics, University of California, San Francisco, San Francisco, CA 94143, USA; 3 Department of Pathology, University of California, San Francisco, San Francisco, CA 94143, USA; 4 Lead contact

## Abstract

Telomerase activation counteracts senescence and telomere erosion caused by uncontrolled proliferation. Epidermal growth factor receptor (EGFR) amplification drives proliferation while telomerase reverse transcriptase promoter (*TERT*p) mutations underlie telomerase reactivation through recruitment of GA-binding protein (GABP). *EGFR* amplification and *TERT*p mutations typically co-occur in glioblastoma, the most common and aggressive primary brain tumor. To determine if these two frequent alterations driving proliferation and immortality are functionally connected, we combine analyses of copy number, mRNA, and protein data from tumor tissue with pharmacologic and genetic perturbations. We demonstrate that proliferation arrest decreases *TERT* expression in a GABP-dependent manner and elucidate a critical proliferation-to-immortality pathway from EGFR to *TERT* expression selectively from the mutant *TERT*p through activation of AMP-mediated kinase (AMPK) and GABP upregulation. EGFR-AMPK signaling promotes telomerase activity and maintains telomere length. These results define how the tumor cell immortality mechanism keeps pace with persistent oncogene signaling and cell cycling.

## In brief

TERT promoter mutations are common in human cancer and confer cellular immortality. McKinney et al. describe the interaction between TERT promoter mutations, EGFR amplification, and the cell cycle in glioblastoma. The results demonstrate how proliferation drivers cooperate with telomere maintenance mechanisms to counteract telomere shortening caused by unlimited cell division.

## Graphical abstract

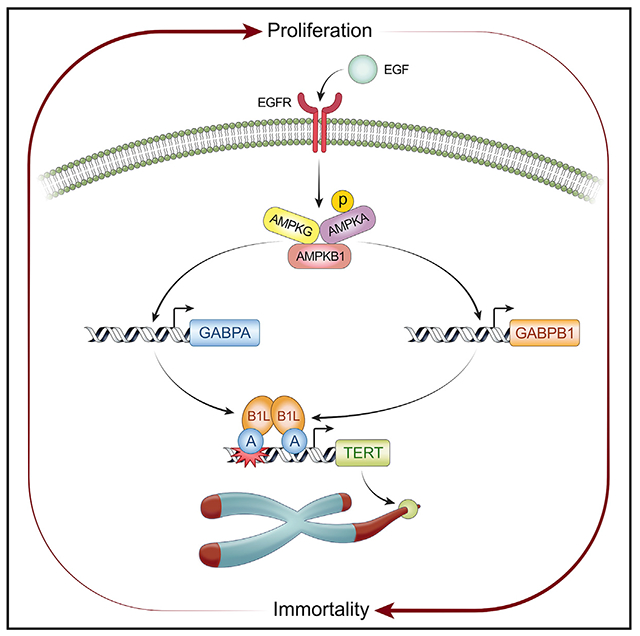

## INTRODUCTION

Telomerase maintains telomere length and integrity in dividing cells (Counter et al., 1992; Greider and Blackburn 1985; Bodnar et al., 1998), but it is unclear how this mechanism adjusts for increasing proliferation in tumor cells. To divide indefinitely, cancer cells must overcome replicative senescence induced by telomere attrition. Ninety percent of cancers do so by reactivating telomerase (Kim et al., 1994; Shay and Bacchetti 1997). In many common types of cancer, telomerase is reactivated through a mutation in the promoter of telomerase reverse transcriptase (*TERT*). *TERT* promoter (*TERT*p) mutations are the most common non-coding mutations in cancer, including the majority of IDH1 wild-type (*IDH1*-WT) primary glioblastoma (GBM) (Huang et al., 2013; Horn et al., 2013; Remke et al., 2013; Quaas et al., 2014; Zehir et al., 2017; Killela et al., 2013; Aquilanti et al., 2021; Vinagre et al., 2013; Arita et al., 2013; Patel et al., 2020). The most common of these mutations, C228T and C250T, which are mutually exclusive (Killela et al., 2013; Zehir et al., 2017) with rare exceptions (Ishi et al., 2021), create a binding site for the E26 transformation-specific (ETS) family of transcription factors (Huang et al., 2013; Bell et al., 2015). GA-binding protein (GABP) is thus far the sole ETS transcription factor that binds to the ETS site created by the mutation and reactivates the *TERT*p (Bell et al., 2015; Stern et al., 2015). Genetic modification of GABP by CRISPR-mediated insertions and deletions led to a reversal of cellular immortality *in vitro* and decreased tumor growth *in vivo* (Mancini et al., 2018). Meanwhile, using an inducible system *in vivo*, we showed that GABP reduction combined with temozolomide, a standard of care for patients with GBM, decreased cellular proliferation and dramatically prolonged survival of mice bearing *MGMT* methylated GBM, highlighting the therapeutic potential of targeting the GABP-*TERT* axis (Amen et al., 2021).

In rapidly dividing tumor cells, the *TERT*p mutation alone may not be sufficiently activating to maintain telomere length (Chiba et al., 2015). Telomerase is expressed in proliferating but not quiescent cancer cells, with levels remaining constant throughout the cell cycle (Holt et al. 1996, 1997; Kyo et al., 1997). Although some oncogenes have been shown to drive *TERT* expression from the WT promoter (Maida et al., 2002; Hoffmeyer et al., 2012; Bermudez et al., 2008; Kyo et al., 1999; Cha et al., 2008; Hsu et al., 2015; Ohba et al., 2016), the mechanisms by which their proliferation signals influence *TERT* expression, particularly from the mutant *TERT*p, are unclear. BRAFV600E signaling leads to *TERT* upregulation in a *TERT*p mutation-specific manner (Gabler et al., 2019; Vallarelli et al., 2016; Liu et al., 2018; Li et al., 2016); however, the BRAFV600E mutation is rare in *TERT*p-mutant GBM.

Epidermal growth factor receptor (EGFR) is activated in 57% of GBM through mutation, structural rearrangement, and/or focal amplification (Brennan et al., 2013). *EGFR* amplification or *TERT*p mutations are indicators of poor prognosis in GBM (Simon et al., 2015; Eckel-Passow et al., 2015; Labussiére et al., 2014; Wijnenga et al., 2017; Aibaidula et al., 2017; Spiegl-Kreinecker et al., 2015). Preliminary studies have shown that 90%–100% of *EGFR*-amplified GBM also have *TERT*p mutations, suggesting a potential cooperative role in driving tumorigenesis (Killela et al., 2013; Labussiére et al., 2014; Aibaidula et al., 2017). Given this striking association, we explored whether signaling from EGFR, a strong driver of cell proliferation, might simultaneously contribute to mutant *TERT*p regulation and associated cell immortality phenotypes in GBM.

## RESULTS

### Proliferation stimulates *TERT* expression in a GABP-dependent manner

Telomerase activity is significantly lower in quiescent tumor cells compared with proliferating cells. Elucidating the specific molecular pathways connecting proliferation to *TERT* upregulation and telomerase activity could inform therapeutic opportunities (Holt et al., 1997). We first determined if the relationship between proliferation and telomerase activity observed in tumor cell lines is also observed in the tumor tissue from 31 cancer types profiled in The Cancer Genome Atlas (TCGA) project, including *TERT*p WT and *TERT*p-mutant tumors. *TERT* expression was significantly positively associated with *MKI67* expression, a marker of actively cycling cells that is absent in cells in the G0 phase of the cell cycle (Figure S1A). In *IDH1*-WT GBM, the majority of which are *TERT*p-mutant, a similarly striking correlation is observed (Figure S1B).

**Figure 1.**
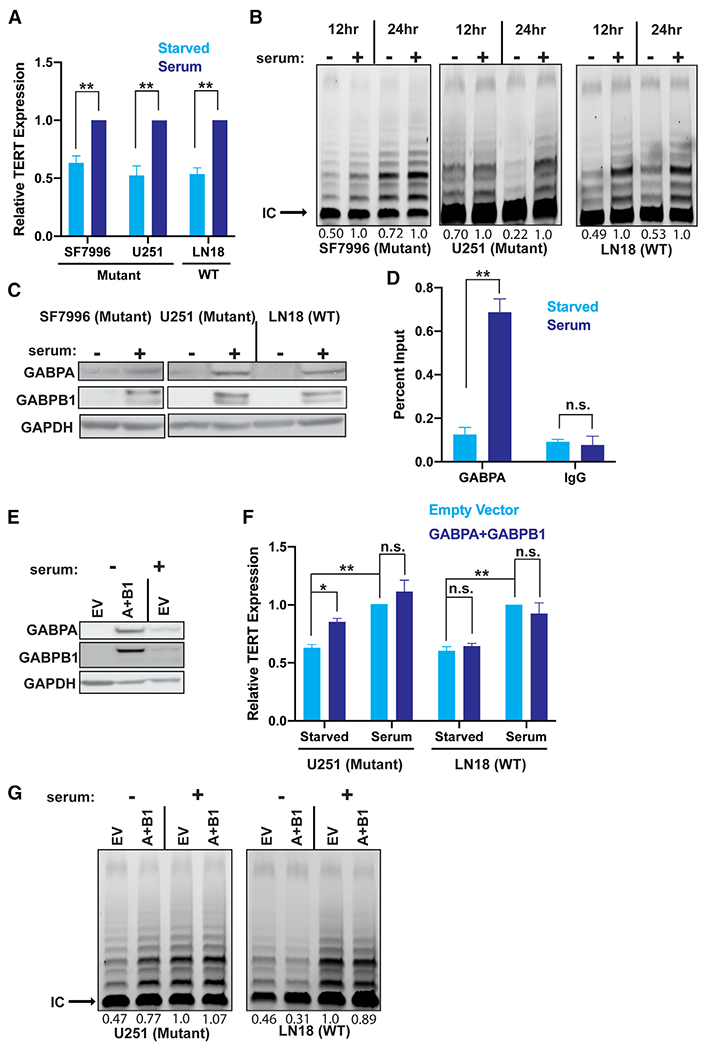
GABP links proliferation to mutant *TERT* promoter activity and telomerase activity (A) *TERT* expression in serum starved *TERT*p-WT and *TERT*p-mut cells after 24 h serum induction, n = 3 biological replicates. (B) Telomerase activity in *TERT*p-WT and *TERT*p-mut cells starved for 12 or 24 h compared with internal control band (IC). (C) Immunoblots in serum starved *TERT*p-WT and *TERT*p-mut cells after 24 h serum induction. (D) GABPA chromatin immunoprecipitation polymerase chain reaction for the *TERT*p in serum starved U251 cells after 24 h serum induction, n = 2. (E) Immunoblots in U251 cells upon serum starvation with or without expression of ectopic *GABPA* and *GABPB1*. (F) *TERT* expression in *TERT*p-mut (left) and *TERT*p-WT (right) cells expressing ectopic *GABPA* and *GABPB1* with or without serum starvation, n = 3 biological replicates. (G) Telomerase activity in *TERT*p-mut (left) and *TERT*p-WT (right) cells expressing ectopic *GABPA* and *GABPB1* with or without serum starvation compared with the IC. (A–F), Student’s t-tests, two-tailed. *p < 0.05, **p < .005, data represent mean ± standard error of the mean. n.s., non-significant.

These data suggest that proliferation and *TERT* expression are correlated in *TERT*-expressing tumors with either a WT or mutant *TERT*p. However, because the mutation rewires *TERT* transcriptional regulation by recruiting GABP, the pathway connecting proliferation to *TERT* expression may differ in WT and *TERT*p-mutant tumors. We first tested for effects of proliferation specifically on the mutant *TERT*p by arresting proliferation in GBM cells (Figure S1C). After 1 day in serum starvation conditions, *TERT* expression and telomerase activity were decreased in a *TERT*p-mutant GBM cell line, a *TERT*p-mutant patient-derived GBM culture, and a *TERT*p WT cell line (Figures 1A and 1B). To test if the connection between proliferation and mutant *TERT*p regulation is more universal, we serum starved 13 cell lines representing the seven most common *TERT*p-mutant cancers and observed decreased *TERT* expression in 12 of them (Figure S1D).

It is not clear how serum starvation causes the decrease in *TERT* across cancer types. In *TERT*p-mutant tumors, GABP plays a central role in *TERT* regulation, and in mouse embryonic fibroblasts, *Gabpa* and *Gabpb1* are expressed at lower levels in serum-starved cells compared with serum-stimulated cells (Yang et al., 2007). We therefore hypothesized that serum starvation-induced changes in GABPA and GABPB1 could underlie the relationship between cell proliferation and the regulation of *TERT*, specifically in *TERT*p-mutant human cancer cells. Indeed, GABPA and GABPB1 expression were decreased upon serum starvation in both *TERT*p-mutant and *TERT*p WT GBM cells. Chromatin immunoprecipitation experiments revealed that the overall decrease in GABP levels was accompanied by a specific reduction of GABP occupancy at the *TERT*p in *TERT*p-mutant GBM cells (Figures 1C and 1D).

Serum starvation induces numerous cellular and molecular changes, many of which could contribute to *TERT* reduction. We, therefore, sought to determine if *GABPA* and *GABPB1* expression is sufficient to rescue *TERT* expression and/or proliferation upon serum starvation. We expressed ectopic *GABPA* and *GABPB1* simultaneously and observed a rescue of basal *TERT* expression and telomerase activity in *TERT*p-mutant cells despite serum starvation (Figures 1F–1G). In contrast, *TERT* expression did not increase significantly upon ectopic expression of *GABPA* and *GABPB1* in cells in serum, suggesting that GABP is not rate limiting in this condition. In contrast with the clear rescue of *TERT* expression, ectopic expression of *GABPA* and *GABPB1* did not restore proliferation to serum-starved cells (Figure S1E). Therefore, the rescue of *TERT* expression is not an effect of increased proliferation. Importantly, rescue of *TERT* expression and telomerase activity by *GAPBA* and *GABPB1* was not observed in *TERT*p WT cells, consistent with GABP having no role in regulating the WT *TERT*p (Figure 1G). Thus, GABP links cell proliferation and *TERT* expression selectively in *TERT*p-mutant tumor cells, providing a partial mechanistic explanation for the long-standing observation that cell proliferation state is associated with telomerase activity levels across a wide spectrum of human cancers.

### *EGFR* amplification and EGFR activity are associated with elevated *TERT* expression

Previous studies have identified a significant co-occurrence of *EGFR* amplification and *TERT*p mutation in two cohorts of 51 and 395 *IDH1*-WT GBM. In contrast, there was no significant co-occurrence between *TERT*p mutation and two other classical GBM alterations, *TP53* mutation and *CDKN2A* deletion (Killela et al., 2013; Labussiére et al., 2014). We validated this finding using an independent cohort of 265 tumors from the MSK-IMPACT study, finding that 94% of *EGFR*-amplified samples harbor *TERT*p mutations (Figure S2A) (Zehir et al., 2017). In contrast, fewer than 1% (1/110) of *EGFR*-amplified samples exhibited *ATRX* loss-of-function mutation indicative of alternative lengthening of telomeres (Figure S2B). Thus, *EGFR* amplification occurs in a significantly higher proportion of *TERT*p-mutant tumors compared with tumors lacking the *TERT*p mutation.

**Figure 2.**
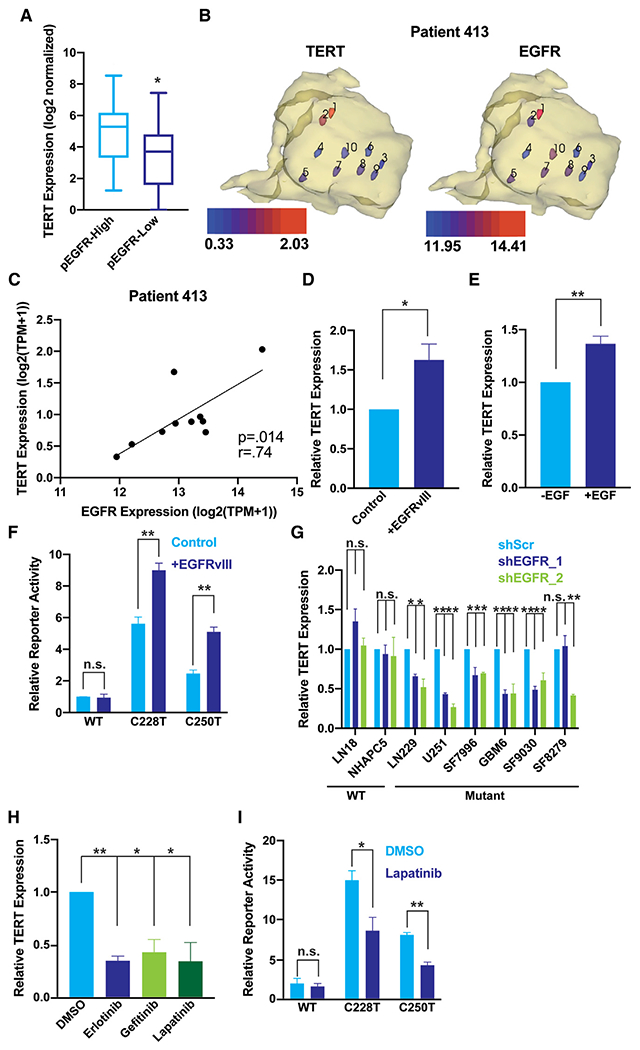
EGFR signaling selectively upregulates the mutant *TERT*p (A) *TERT* mRNA expression in *IDH1*-WT GBM with different EGFR expression. Tumors profiled by TCGA with available RNA-Seq and RPPA data, and tumor purity greater than 60%, were stratified into 27 EGFR-high, 27 EGFR-low cases by RPPA expression. Whiskers represent 5 th and 95 th percentile values. Wilcoxon rank-sum test, two-tailed *p < 0.05, **p < .005. (B) log2-normalized mRNA expression from 10 intratumoral, spatially mapped samples for *TERT* (left) and *EGFR* (right) in the 3D context of the tumor (yellow, tumor derived from T2 MRI). (C) Correlation of EGFR and *TERT* expression within 10 intratumoral samples. r^2^, Pearson correlation coefficient. (D) *TERT* expression measured by reverse transcriptase quantitative PCR after 12 h doxycycline induction of EGFRvIII in serum starved U251 cells. Data are normalized relative to serum starved cells (-doxycycline) within each replicate, n = 3 biological replicates. (E) *TERT* expression measured by reverse transcriptase polymerase chain reaction (RT-qPCR) after 12 h EGF induction in serum starved U251 cells. Data are normalized relative to the serum starved (-EGF) condition within each replicate, n = 3 biological replicates. (F) *TERT*p-luciferase reporter assays after 12 h of EGFRvIII induction in serum starved U251 cells. Data are normalized relative to WT reporter activity in serum starved cells (-EGFRvIII induction) within each replicate, n = 3 biological replicates. (G) *TERT* expression after 72 h of shRNA targeting EGFR, measured by RT-qPCR in *TERT*p-WT and *TERT*p-mut cells. Data are normalized relative to scrambled control (shScr) for each cell line, n = 3 biological replicates. (H) *TERT* expression after 72 h of treatment of GBM6 cells with 1uM EGFR inhibitor, measured by RT-qPCR. Data are normalized relative to DMSO within each replicate, n = 3 biological replicates. (I) *TERT*p-luciferase reporter assays in LN229 cells after 72 h of pharmacological EGFR inhibition. Data are normalized relative to DMSO treated WT reporter, n = 3 biological replicates. (D–I), Student’s t-tests, two-tailed. *p < 0.05, **p < .005, data represent mean ± standard error of the mean, n.s., non-significant.

To explore the association between the mutant *TERT*p and EGFR beyond the genomic level, we compared their expression at the mRNA and protein levels in multiple GBM datasets. First, in the 129 *IDH1*-WT GBM from TCGA, *TERT* expression is significantly higher in tumors with an *EGFR* amplification compared with those without (Figure S2C). Second, stratifying the 54 TCGA GBM with reverse phase protein array (RPPA) data by their level of EGFR activation, as measured by phosphorylation of EGFR Y1068 (Rojas et al., 1996), shows that a high level of EGFR activation is associated with greater *TERT* expression (Figure 2A). As a complementary approach to the large TCGA cohort with single samples per patient, we examined cases from our UCSF cohort with a relatively large number of samples per tumor. Unlike the TCGA cohort, intratumoral comparisons control for the strong, potentially confounding differences in genetic background between different patients. We selected four GBM cases with a total of 33 spatially mapped samples (7–11 samples per patient) to maximally sample intratumoral heterogeneity, which is essential for correlation analysis. Exome sequencing data were used to estimate tumor purity and detect EGFR amplification. RNA sequencing data was analyzed for EGFR and TERT expression level (Figure 2B and Videos S1 and S2). Despite high tumor cell content in all samples, the level of *EGFR* amplification and expression was heterogenous among samples from the same patient. Within each case, *EGFR* expression positively correlated with *TERT* expression (Figures 2B, 2C, and S2D). Importantly, the sample from patient 454 with the lowest *TERT* expression had no detectable *EGFR* amplification. From these inter- and intratumoral datasets, we conclude that *EGFR* amplification and expression are associated with elevated *TERT* expression in *TERT*p-mutant tumors.

### EGFR selectively regulates the mutant *TERT*p

Given the significant associations between elevated *EGFR* mRNA and protein phosphorylation levels, *TERT*p mutation and *TERT* expression in tumor tissue from patients with GBM, we tested for a potentially causal relationship using three independent experimental approaches. First, we used EGFRvIII, a common structural variant detected in approximately 20% of GBM overall and approximately one-half of the GBM cases with amplified *EGFR* (Brennan et al., 2013). EGFRvIII, which exhibits constitutive ligand-independent activity (Batra et al., 1995; Huang et al., 1997), was expressed in an inducible manner in *TERT*p-mutant U251 cells and resulted in a rapid and significant increase in *TERT* expression (Figure 2D). Second, we stimulated endogenous EGFR with EGF ligand, which also caused a significant increase in *TERT* expression (Figure 2E). These studies support a causal relationship, but cannot definitively show selectivity for the mutant promoter since the WT promoter is not active in these cells. Therefore, our third approach used WT and mutant *TERT*p luciferase promoter assays. EGFRvIII overexpression drove *TERT*p activity to a much higher level and in a mutation-specific manner, increasing reporter activity from a promoter with either the C228T or C250T mutation while not significantly altering activity of the WT *TERT*p (Figure 2F). To test if inhibiting steady-state levels of endogenous EGFR alters *TERT* expression, we treated four GBM cell lines and four GBM patient-derived lines (Fouse et al., 2014; Sarkaria et al., 2006) with three distinct EGFR inhibitors or with two distinct short hairpin RNAs (shRNAs) targeting *EGFR*. Whether by pharmacologic or genetic means, decreasing EGFR activity in cell lines with *TERT*p mutation consistently decreased *TERT* expression, with only two exceptions (lapatinib in SF7996 and shEGFR-1 in SF8279, Figure S3A and 2G), an effect not seen in *TERT*p WT lines (Figures 2G and S3A). Furthermore, shRNA knockdown or pharmacologic inhibition of EGFR in a natively *EGFR*-amplified patient-derived cell culture, GBM6, also resulted in a significant decrease in *TERT* expression (Figures 2G and 2H). The exclusivity of these *TERT* responses to EGFR inhibition in cell lines with the *TERT*p mutation suggests EGFR signaling specifically activates the mutant promoter. To test the specificity for the mutant *TERT*p in an isogenic background, we compared the effect of EGFR inhibition on luciferase activity in LN229 cells with either the WT or mutant *TERT*p driving luciferase. These experiments demonstrate that EGFR inhibition regulates the C228T and C250T mutant *TERT*p, while not affecting activity of the WT promoter (Figure 2I). We conclude that EGFR activation selectively stimulates expression of the mutant *TERTp* in GBM cells. Thus, in addition to its role in stimulating cell proliferation, EGFR may also reinforce the immortal state of GBM cells via the upregulation of *TERT*. Determining how EGFR upregulates *TERT* specifically from the mutant *TERT*p is, therefore, critical for understanding the potential interaction between oncogene signaling, cell proliferation, and tumor cell immortality at the molecular level.

### EGFR regulates GABP expression

The two most common *TERTp* mutations, C228T and C250T, create ETS transcription factor binding motifs that recruit the heterotetrameric form of GABP, selectively activating the mutant *TERT*p (Bell et al., 2015; Mancini et al., 2018). Given the specificity of EGFR signaling in regulating the mutant but not WT *TERTp* (Figures 2D–2I), we next sought to determine whether this regulation involves GABP. Using the same stratification of TCGA tumors into phosphorylated EGFR (pEGFR)-high and pEGFR-low groups (Figure 2A), we found that the average *GABPB1* expression was significantly increased in EGFR-high tumors, and *GABPA* mRNA showed a trend toward being elevated (Figures S4A and S4B).

**Figure 3.**
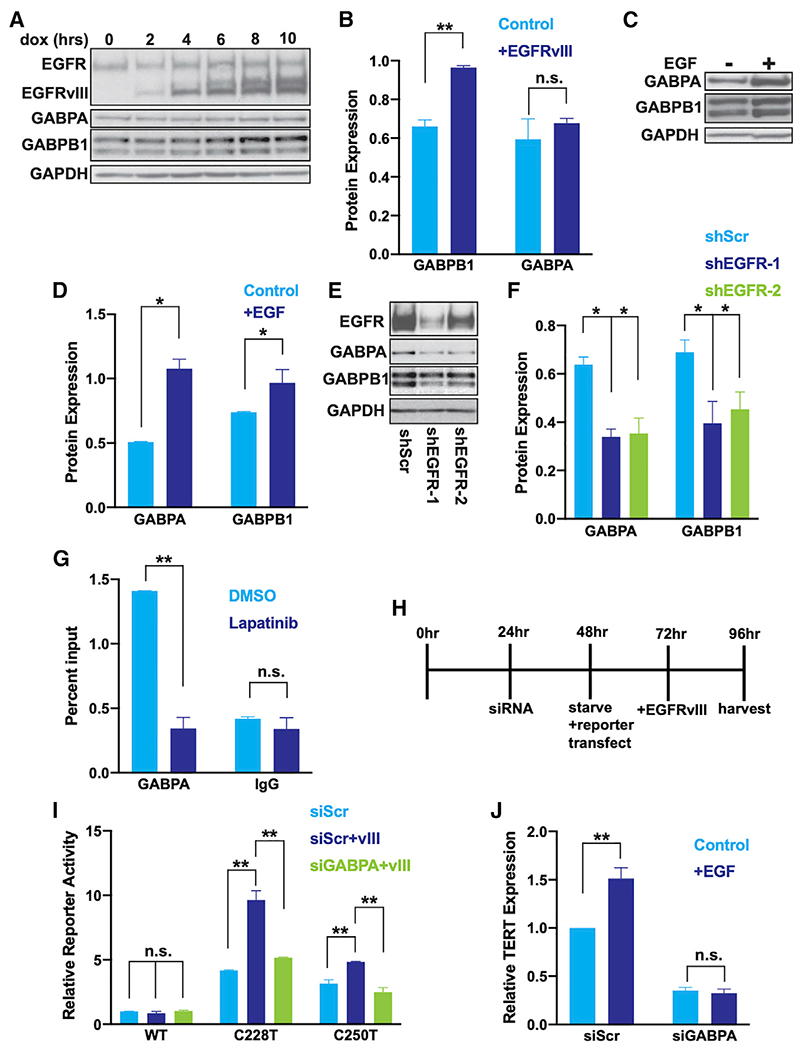
EGFR signaling upregulates the mutant *TERT*p by increasing GABP expression (A) Representative immunoblots of EGFR, GABPA, and GABPB1 after EGFRvIII induction in U251 cells. (B) Quantification of the 10-h time point of immunoblots from (A), n = 3 biological replicates. (C) Immunoblots of GABPA and GABPB1 after 12 h of EGF induction in serum starved LN229 cells. (D) Quantification of immunoblots from (C), n = 3 biological replicates. (E) Immunoblots of EGFR, GABPA, and GABPB1 after 72 h of shRNA targeting EGFR in LN229 cells. (F) Quantification of immunoblots from (E). (G) GABPA and IgG isotype control chromatin immunoprecipitation (ChIP) reverse transcriptase polymerase chain reaction (qPCR) for the *TERT*p after 72 h pharmacological EGFR inhibition in LN229 cells, n = 2 biological replicates. (H) Experimental timeline of EGFRvIII induction and luciferase reporter assay after serum starvation, for data in (I) and (J). (I) *TERT*p-luciferase reporter activity in U251 cells treated for 72 h with siRNAs targeting *GABPA* or scrambled control (siScr). Data are normalized relative to serum-starved U251 cells with siScr, WT reporter activity within each replicate, n = 3 biological replicates. (J) *TERT* expression measured by RT-qPCR after a 12-h EGF induction in serum-starved cells that were also treated with siRNAs for 72 h targeting *GABPA* or siScr control in LN229 cells. Data are normalized relative to serum starved cells (−EGF) within each condition, n = 3 biological replicates. (B–G, I–J) Student’s t-tests, two-tailed. *p < 0.05, **p < .005, data represent mean ± standard error of the mean, n.s., non-significant.

The statistically significant association between EGFR phosphorylation and GABP expression in tumor tissue (Figures S4A and S4B) led us to hypothesize that EGFR activation of the mutant *TERTp* is mediated by transcriptional upregulation of one or more GABP subunits. To test this hypothesis, we measured GABPA and GABPB1 after modulating EGFR signaling. Using the inducible EGFRvIII, we found that EGFRvIII rapidly increases GABPB1 expression (Figures 3A and 3B). EGF stimulation in *TERT*p-mutant GBM cells also increased expression of both GABPA and GABPB1 (Figures 3C and 3D). Conversely, shRNA-mediated knockdown of *EGFR* as well as pharmacologic inhibition of EGFR decreased GABPA and GABPB1 expression (Figures 3E, 3F, S3C, and S3D). Cumulatively these data define the connections between EGFR signaling and GABP in *TERT*p-mutant GBM.

We next asked whether EGFR regulates *TERT* expression through GABP. When we inhibited EGFR pharmacologically, the reduced GABP expression was accompanied by a decrease in GABP occupancy at the mutant *TERT*p (Figure 3G). Furthermore, transient knockdown of GABP expression reduced EGFRvIII-stimulated activity of the mutant *TERT*p and reduced EGF-stimulated *TERT* expression (Figures 3H–3J). In contrast, there was no change in activity of the WT *TERT*p (Figure 3I). Together these results implicate EGFR signaling in the regulation of GABP subunit expression and subsequently *TERT* regulation via direct GABP binding to the mutant *TERT*p.

**Figure 4.**
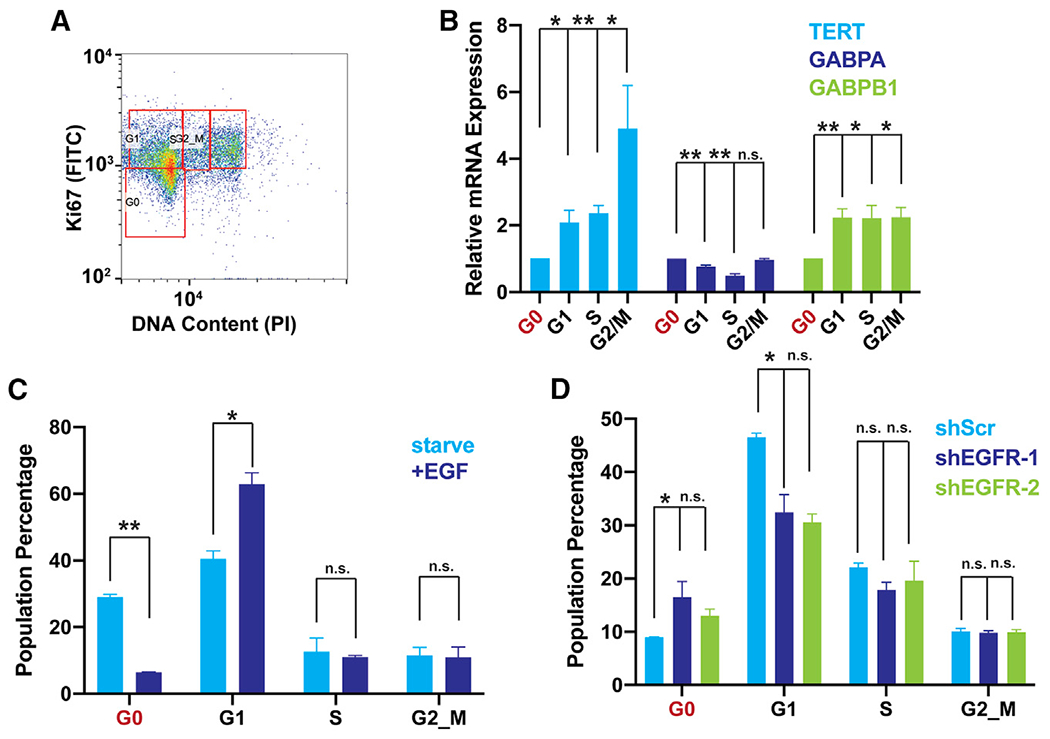
EGFR signaling induces entry into the cell cycle and elevated GABP and TERT (A) Representative flow cytometry plot of cell cycle phases (G0, G1, S, G2_M) based on DNA content (propidium iodide [PI]) after 24 h of starvation and 12 h EGF induction and cell cycling measured by Ki67 expression. (B) *TERT* expression in phases of the cell cycle (G0, G1, S, G2_M) measured by reverse transcriptase polymerase chain reaction in U251 cells sorted as in (A), n = 3 biological replicates. (C) Percentage of cells in phases of the cell cycle (G0, G1, S, G2_M) after 24 h starvation and 12 h of EGF induction, measured by flow cytometry of PI and KI67, n = 3 biological replicates. (D) Percentage of cells in phases of the cell cycle (G0, G1, S, G2_M) after shRNA targeting of EGFR, measured by flow cytometry of PI and KI67. (B–D) Student’s t-tests, two-tailed. *p < 0.05, **p < .005, data represent mean ± standard error of the mean, n.s., non-significant.

EGFR is a driver of cell cycling and proliferation in GBM and other tumor types. To distinguish cell cycle-related and unrelated effects on *TERT* and GABP expression, we analyzed *TERT* and GABP subunit expression in cells that were serum starved and then induced by EGF into different phases of the cell cycle (G0, G1, S, and G2_M). In contrast with *GABPA, TERT* and *GABPB1* were lower in non-cycling (G0) cells relative to cells in G1, S, and G2_M (Figures 4A and 4B). We next determined cell cycle proportions after EGF induction or shRNA knockdown of EGFR. We observed a decrease in G0 cells after EGF stimulation and a slight increase in G0 cells after EGFR knockdown and concomitant changes in G1, suggesting EGFR signaling promotes entry into the cell cycle (Figures 4C and 4D). Therefore, consistent with our hypothesis, EGFR promotes entry into the cell cycle and elevated *GABPB1* and *TERT*. However, *GABPA* is not increased in cells entering the cell cycle and, therefore, EGFR exerts a cell cycle-independent effect on *TERT* regulation as well.

### AMP-mediated kinase regulates the GABP-TERT axis downstream of EGFR

**Figure 5.**
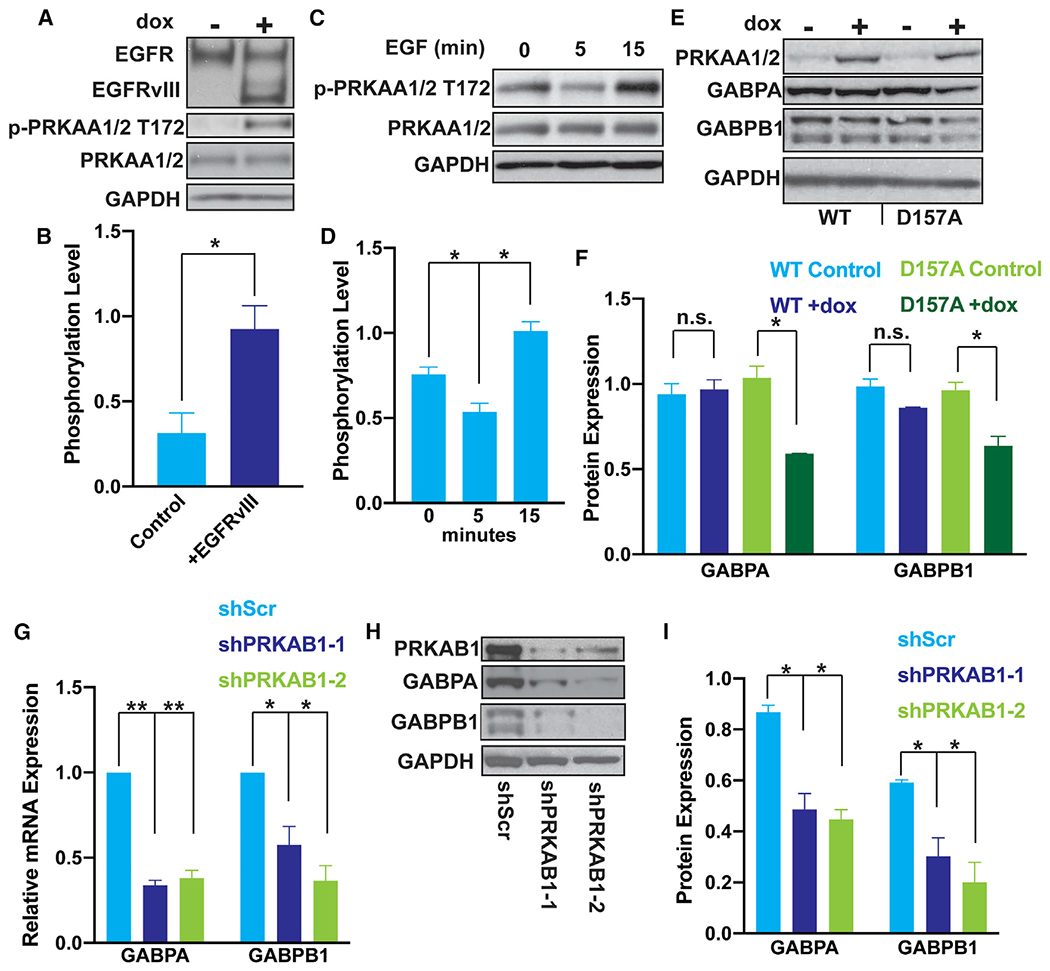
Activated AMPK upregulates GABP subunit expression downstream of EGFR (A) Representative immunoblots of EGFR, p-PRKAA1/2 T172 and AMPKα after doxycycline induction of EGFRvIII in U251 cells. (B) Quantification of immunoblots from (A). Data are expressed as ratio of p-PRKAA1/2 to total PRKAA1/2 within each replicate, n = 3 biological replicates. (C) Representative immunoblots of p-PRKAA1/2 T172 and PRKAA1/2 upon EGF induction in serum-starved LN229 cells. (D) Quantification of immunoblots from panel C. Data are expressed as the ratio of p-PRKAA1/2 to total PRKAA1/2 within each replicate, n = 3 biological replicates. (E) Representative immunoblots of PRKAA1/2, GABPA, and GABPB1 upon doxycycline-induction of PRKAA1/2 (WT) or catalytically dead PRKAA1/2 (D157A) expression in LN229 cells. (F) Quantification of immunoblots from (E), n = 3 biological replicates. (G) *GABPA* and *GABPB1* mRNA expression upon shRNA-mediated knockdown of *PRKAB1* in LN229 cells, measured by reverse transcriptase polymerase chain reaction, n = 3 biological replicates. (H) Immunoblots of GABPA, GABPB1, and PRKAB1 after 72 h shRNA-mediated *PRKAB1* knockdown in LN229 cells. (I) Quantification of immunoblots from (H), n = 3 biological replicates. (A–I) Student’s t-tests, two-tailed. *p < 0.05, **p < .005, data represent mean ± standard error of the mean, n.s., non-significant.

Multiple pathways downstream of EGFR could play a role in EGFR regulation of GABP and *TERT*. One downstream mediator of EGFR signaling of special interest is AMP-mediated kinase (AMPK), a heterotrimeric complex composed of α, β, and γ subunits that plays a key role in energy sensing and cellular metabolism. AMPK has been described as tumorigenic and tumor suppressing in tissue-specific contexts and in different cellular energy states (Ríos et al., 2013; Faubert et al., 2013; Shaw et al., 2004; Inoki et al., 2006; Jones et al., 2005). Although AMPK has been most widely studied as a metabolic regulator, two studies report a distinct role as a stimulator of proliferation in glioma (Ríos et al., 2013; Chhipa et al., 2018). AMPK signaling is overactive in GBM compared with normal brain. In two GBM cell lines, AMPK drives tumorigenesis in part through the transcriptional upregulation of *GABPA* (Chhipa et al., 2018), though it has no reported role in the regulation of *GABPB1*. AMPK regulates oncogenic signaling downstream of EGFR overexpression and EGF stimulation in other cancer contexts, suggesting AMPK as a potential link between EGFR and GABP regulation (Han et al., 2018; Katreddy et al., 2018). In RPPA data from GBM, the level of activating phosphorylation of threonine 172 on PRKAA1 (AMPKA1_PT172) (Stein et al., 2000) is significantly higher in pEGFR-high tumors than pEGFR-low tumors, further supporting that EGFR signaling and AMPK signaling are associated in GBM *in vivo* (Figure S4C). To determine if the strong statistical association of EGFR-AMPK activity in patient tumor tissue reflects a functional relationship, we stimulated GBM cells with EGF or overexpressed EGFRvIII. With either approach to activating EGFR signaling, AMPK activation, as determined by phosphorylation of T172, was increased (Figures 5A–5D).

With a firm connection established between EGFR signaling and AMPK activity in GBM tissue and across multiple cell lines, we turned our attention to the question of how AMPK signaling may in turn influence regulation of the mutant *TERT*p. The PRKAB1 subunit of the AMPK complex is overexpressed in GBM and is essential for kinase activity of AMPK and downstream signaling in GBM (Chhipa et al., 2018). Our analysis of TCGA RNA sequencing data demonstrated a strong correlation between expression of the AMPK subunit *PRKAB1* and both *GABPA* and *GABPB1* (Figures S5A and S5B). The correlation with *GABPB1* was not expected because unlike the ETS factor gene *GABPA, GABPB1* is a Notch-Ankyrin repeat family member and has distinct regulation. Therefore, we again took three independent approaches to determine if AMPK signaling regulates transcription of one or both GABP subunits. First, we overexpressed dominant negative PRKAA2, which disrupts AMPK signaling (Chhipa et al., 2018), and observed that GABPA and GABPB1 expression was decreased (Figures 5E and 5F). Second, we used shRNA knockdown of the AMPK regulatory subunit *PRKAB1*, which also decreased GABPA and GABPB1 mRNA and protein (Figures 5G–5I). Third, we generated three full gene knockout clones of *PRKAB1* using guides flanking the entire mRNA-coding portion of the gene. *TERT* mRNA expression and telomerase activity, as well as *GABPA* and *GABPB1* mRNA and protein expression were all consistently reduced in the three knockout clones (Figures S6A–SD). Unlike *EGFR* knockdown, knockdown of *PRKAB1* did not cause a substantial shift in cycling to non-cycling cells, demonstrating that AMPK signaling acts downstream of EGFR (Figures 5A–5F), but is decoupled from cell cycle changes in this time frame (Figure S7A). Given the consistent results of these orthogonal approaches, we conclude that AMPK positively regulates both *GABPA* and *GABPB1*. These data delineate the core components of a molecular pathway connecting EGFR and regulation of the mutant *TERT*p.

**Figure 6.**
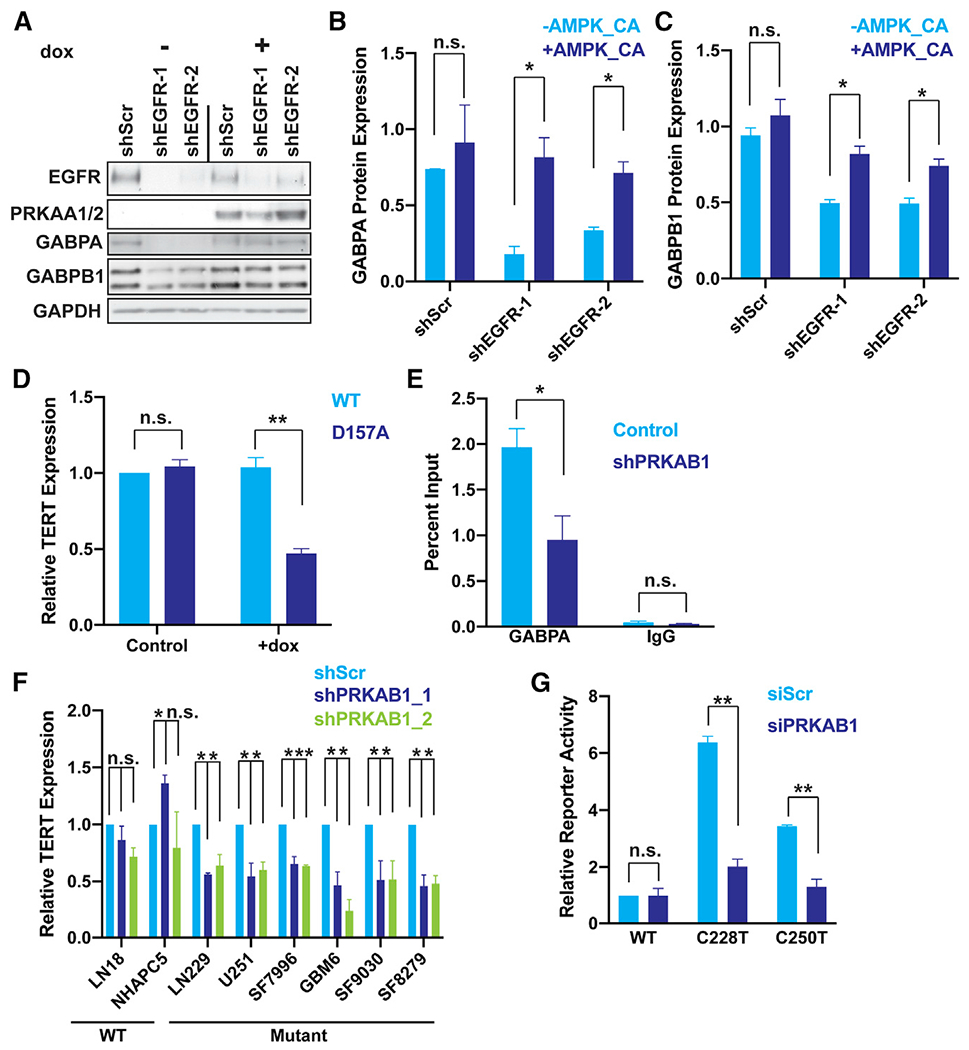
AMPK signaling selectively regulates the mutant *TERT*p (A) Representative immunoblots of EGFR, PRKAA1/2, GABPA, GABPB1, and GAPDH after shRNA knockdown of EGFR and 72 h induction of constitutively active PRKAA1. (B and C) Quantification of (A), n = 3 biological replicates. (D) *TERT* expression measured by reverse transcriptase polymerase chain reaction (RT-qPCR) after 72-h doxycycline-induction of PRKAA2 (WT) or catalytically dead PRKAA2 (D157A) expression in LN229 cells. Data are normalized relative to uninduced WT AMPKA-vector containing cells within each replicate, n = 3 biological replicates. (E) GABPA chromatin immunoprecipitation quantitative polymerase chain reaction for the *TERT*p after 72 h doxycycline-inducible shRNA-mediated *PRKAB1* knockdown in LN229 cells. IgG isotype was used as a control. Data are expressed as a percentage of genomic DNA input, n = 2 biological replicates. (F) *TERT* expression upon shRNA-mediated knockdown of *PRKAB1* measured by RT-qPCR in *TERT*p-WT and *TERT*p-mut cell lines and patient-derived cultures. Data are normalized relative to scrambled control (shScr) control, n = 3 biological replicates. (G) *TERT*p-luciferase reporter assays after 72-h siRNA-mediated knockdown of *PRKAB1* for WT, C228T, and C250T promoters. Data are normalized relative to siRNA scramble control *TERT*p WT reporter in LN229 cells, n = 3 biological replicates. (B–G) Student’s t-tests, two-tailed. *p < 0.05, **p < .005, data represent mean ± standard error of the mean, n.s., non-significant.

We next wanted to show that EGFR regulates GABP through AMPK. To do this, we inducibly overexpressed a constitutively active PRKAA1 variant in the context of EGFR knockdown. AMPK activity rescued GABPB1 and GABPA expression after EGFR shRNA-mediated downregulation (Figures 6A–6C), showing that EGFR regulates GABPA and GABPB1 expression through AMPK. As a second approach, we stimulated PRKAB1 knockout (KO) cells with EGF and observed no increase in *GABPA* and a slight increase in *GABPB1* (Figures S6E and S6F). The AMPK-mediated increase in GABP subunit expression could potentially drive increased *TERT* expression. Therefore, we next measured *TERT* expression, *TERT*p activity, and GABP occupancy of the mutant *TERT*p following AMPK modulation. Along these lines, overexpression of dominant-negative PRKAA2 decreased *TERT* expression (Figure 6D). Similarly, knockdown of *PRKAB1* reduced *TERT* expression, reduced promoter activity in a mutation-specific manner, and decreased binding of GABP to the mutant *TERT*p (Figures 6E–6G). Stimulation of *PRKAB1* KO clones with EGF did not increase *TERT* expression, suggesting that EGF-induced GABP expression requires AMPK (Figure S6G). Downstream of EGFR, AMPK signaling therefore regulates the mutant *TERTp* through GABP subunit modulation.

### EGFR-AMPK signaling cooperates with *TERTp* mutations to maintain telomere length

**Figure 7.**
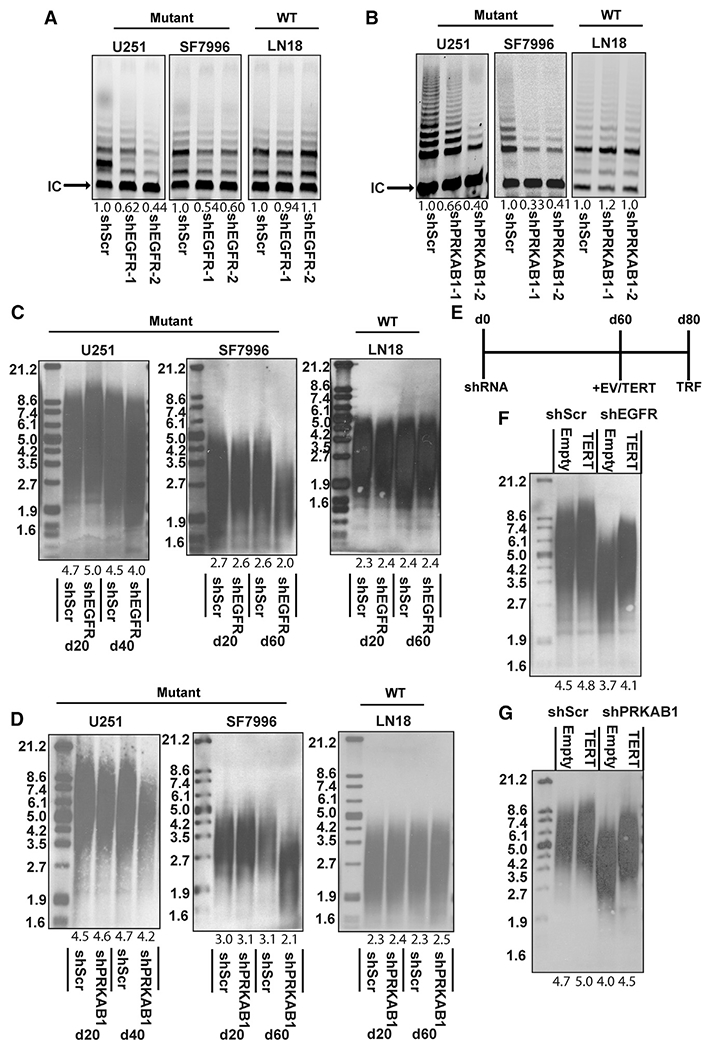
The EGFR-AMPK axis regulates telomerase activity and telomere length in TERT promoter mutant GBM (A) Telomerase activity after 6 days of shRNA-mediated knockdown of EGFR in *TERT*p-mut and *TERT*p-WT cells compared to internal control band (IC). (B) Telomerase activity after 6 days of shRNA-mediated knockdown of PRKAB1 in *TERT*p-mut and *TERT*p-WT cells compared with the internal control band (IC). (C) Telomere length assessed by telomere restriction fragmentation after shRNA-mediated knockdown of PRKAB1 in *TERT*p-mut and *TERT*p-WT cells. (D) Telomere length assessed by telomere restriction fragmentation after shRNA-mediated knockdown of PRKAB1 in *TERT*p-mut and *TERT*p-WT cells. (E) Timeline of long-term telomere restriction fragmentation *TERT* rescue experiments. (F) Telomere length assessed by telomere restriction fragmentation after shRNA-mediated knockdown of EGFR in U251 followed by rescue with *TERT* coding sequence or empty vector. (G) Telomere length assessed by telomere restriction fragmentation after shRNA-mediated knockdown of PRKAB1 in U251 followed by rescue with *TERT* relative to empty vector control.

The elevated *TERT* expression downstream of EGFR-AMPK signaling may have little functional consequence, or alternatively, it could provide the increased telomerase activity required for telomere maintenance in the rapidly dividing GBM cells. To distinguish between these possibilities, we first knocked down *PRKAB1* and *EGFR* and measured telomerase activity. Either *PRKAB1* or *EGFR* knockdown was sufficient to reduce telomerase activity in *TERT*p-mutant, but not *TERT*p WT, GBM cells (Figures 7A and 7B). In addition to reduced telomerase activity, proliferation slowed slightly in the *EGFR* and *PRKAB1* knockdown cells (Figure S7B). Telomerase activity lengthens telomeres while rapid cell division shortens them. As our data and prior studies suggest, EGFR-AMPK can regulate both of these cancer cell phenotypes. We asked whether knockdown of *PRKAB1* or *EGFR* would lead to telomere attrition in cells that are actively dividing, albeit slightly slower (Figure S7B). Following the cells over a 40- to 60-day period, we observed progressive telomere shortening in *TERT*p-mutant GBM cells with *EGFR* or *PRKAB1* knockdown, but not in *TERT*p WT cells (Figures 7C and 7D). The telomere shortening could be rescued in *PRKAB1* or *EGFR* knockdown cells by ectopic expression of *TERT* (Figures 7E and 7F). Upon *PRKAB1* or *EGFR* knockdown, cells underwent fewer population doublings (Figure S7B), yet their telomeres shortened significantly compared to controls. We conclude that reduced telomerase activity caused by *EGFR* and *PRKAB1* knockdown in actively dividing GBM cells leads to telomere attrition. Thus, EGFR regulation of the mutant *TERT* promoter has phenotypic consequences on telomere regulation.

## DISCUSSION

Tumor cell proliferation and replicative immortality are interdependent hallmarks of human cancer, but very little is known about how they are mechanistically connected. Our study begins to define a major molecular pathway linking proliferation driven by EGFR amplification and overexpression to *TERT*p mutation, two of the most common genetic events in GBM, and demonstrates their cooperation in telomere maintenance. The transcription factor GABP is a central node in this pathway, receiving signals from EGFR through AMPK and selectively activating *TERT* expression, the rate limiting factor in telomerase activity and tumor cell immortality. We demonstrate that the effects of EGFR on GABP-*TERT*p regulation can be partially attributed to the promotion of cell cycling and partially to cycling-independent effects. From the EGFR perspective, our study identifies and delineates a previously unknown arm of the EGFR signaling pathway that regulates the immortality mechanism. Investigation into signaling through other oncogenic pathways that drive GBM could reveal additional links to the mutant *TERTp*. For example, AMPK is also activated by *KRAS* overexpression or *PTEN* knockdown in GBM (Chhipa et al., 2018), and *KRAS* mutation or amplification is found in approximately 3% of GBM, while *PTEN* alteration is in approximately 40% of GBM, including in roughly 50% of EGFR-amplified cases (Brennan et al., 2013). Whether these genetic alterations result in the activation of AMPK-GABP-*TERT* alone, or together with *EGFR* amplification, is currently unknown. Given that serum starvation reduced *TERT* expression in the most common *TERT*p-mutant cancers (Figure S1D), it is possible that other drivers of proliferation regulate *TERT* in different genetic backgrounds. In other cancer types, BRAF signaling has been linked to *TERT*p mutations; therefore, a wider survey of the interaction of oncogenic alterations with *TERT*p mutations and telomere maintenance is warranted.

Drugs targeting telomerase have long been of interest for cancer treatment, but thus far have been unsuccessful in cancer clinical trials. For example, telomerase inhibitors that target the telomerase RNA template TERC, such as imetelstat, showed promise in preclinical studies, including in GBM, but failed in clinical trials in part owing to “on-target” toxicity in normal stem cells with TERC and *TERT* expression (Marian et al., 2010; Chiappori et al., 2015; Kozloff et al., 2010). Our results suggest that targeting EGFR with clinical inhibitors could decrease telomerase activity in a tumor-specific manner, via decreased activity of the mutant *TERT*p. However, the EGFR inhibitor-induced telomerase reduction is unlikely on its own to have a strong and sustained antitumor effect. Future development of combination therapies targeting EGFR to block proliferation and reduce *TERT* while also blocking *TERT* or GABP directly would be of interest.

*TERT*p mutations seem to be critical throughout tumorigenesis for ongoing telomere maintenance, making them an attractive, cancer-specific therapeutic target (Brastianos et al., 2017; Körber et al., 2019). In this regard, we recently proposed GABP as a potential therapeutic target in combination with chemotherapy (Mancini et al., 2018; Amen et al., 2021). Similarly, others have demonstrated that gene editing of the mutant *TERT*p reverses cellular immortality (Li et al., 2020). Identifying drug-gable kinases upstream of the GABP-*TERT* axis such as EGFR and AMPK could facilitate future targeting of telomere maintenance, although this would require testing combinations with additional approaches to reduce *TERT* to a critically low level that is insufficient to maintain telomeres as tumor cells proliferate (Agarwal et al., 2021). EGFR targeting has not yet proven effective in GBM, which may in part be due to intratumoral heterogeneity (Figure S2) (Sottoriva et al., 2013). It is also important to identify approaches that will more rapidly induce telomere dysfunction so there is less dependency on relatively long periods of cell division in the absence of *TERT* for telomere reduction and tumor cell killing.

*TERT*p mutations activate TERT to maintain telomeres in cancer cells, but generally at a shorter length relative to other telomere maintenance mechanisms, such as alternative lengthening of telomeres in *ATRX* mutant glioma. Telomere dysfunction is associated with chromosomal instability, genome reduplication, chromothripsis, and subsequent focal amplifications or deletions (Chin et al., 1999; Davoli et al., 2010; Saretzki et al., 1999). Recent work has suggested that chromosomal abnormalities encompassing the genes driving proliferation, including gain of chromosome 7 where EGFR and PDGFA reside, precede *TERT*p mutations in GBM and that *TERT*p mutations are necessary for clonal expansion (Körber et al., 2019). The early placement of *EGFR* copy number gains and *TERT*p mutations on the evolutionary timescale of GBM development highlights the necessary role of this duet in the classical subtype of *IDH1*-WT GBM. That *TERT*p mutations are necessary for clonal expansion suggests telomere maintenance is also required by pre-cancer cells to overcome replicative senescence after acquiring strong drivers such as EGFR through amplification or copy number gain.

### Limitations of the study

*TERT* is expressed at a very low level, and endogenous protein is difficult to detect with currently available antibodies, which necessitates of the use of the telomerase activity assay. Isogenic cell lines differing only in the *TERT* promoter status that also show a functional difference in tumorigenesis were not available at the time of this study. Thus, our study and others are performed using a range of *TERT* promoter mutant and WT cell lines with different genetic backgrounds. An isogenic model derived from human-induced pluripotent stem cells has been described very recently (Miki et al., 2022) and could be used in future studies to understand genetic dependencies of *TERT* promoter mutant cancers.

## STAR★METHODS

### RESOURCE AVAILABILITY

#### Lead contact

Further information and requests for resources and reagents should be direct to and will be fulfilled by the lead contact, Joseph Costello (joseph.costello@ucsf.edu).

#### Materials availability

This study did not generate any unique reagents.

#### Data and code availability

- TCGA data used for copy number alterations, mRNA expression, and RPPA expression can be publicly accessed (https://www.cancer.gov/about-nci/organization/ccg/research/structural-genomics/tcga).
- Previously uploaded whole exome sequencing and RNA sequencing libraries can be accessed in the European Genome-Phenome Archive:EGA00001003710 and is publicly available at the time of publication. Accession numbers are listed in key resources table.
- This paper does not report original code.
- Any additional information required to reanalyze the data is available from the lead contact upon request.

### EXPERIMENTAL MODEL AND SUBJECT DETAILS

#### Spatially mapped tumor sample collection

University of California, San Francisco’s Institutional Review Board approved sample collection and usage. All patients provided informed written consent prior to sample acquisition. Cases were selected on the basis of an initial sample containing both *TERT*p mutation and *EGFR* amplification identified by the UCSF500 clinical cancer sequencing panel. All available intra-tumoral samples were then tested for *TERT*p mutation by PCR and Sanger sequencing (33/33 samples were positive) and for *EGFR* amplification using the exome sequencing data. Samples from the following patients were used in this study: Patient 413 (male, age 63), patient 454 (female, age 60), patient 498 (female, age 58), and patient 500 (male, age 72).

#### Culturing human tumor cell lines

Cell lines and patient-derived cultures were grown in a 37°C incubator at 5% CO_2_. SF7996 (male), LN229 (female), U251 (male), and LN18 (male) were cultured in DMEM/Ham’s F-12 1:1 supplemented with 10% Hyclone FBS (GE Life Sciences #SH30071) and 1% Pen-Strep (Gibco #15140-122). NHAPC5 were grown in DMEM (Corning # 10-013-CV) supplemented with 10% FBS and 1% Pen-Strep as previously described (Mancini et al., 2018; Amen et al., 2021). GBM6 (male) tumor-sphere cultures (Sarkaria et al., 2006) were cultured on ultra low attachment plates (Corning #CLS3471) in sterile-filtered Neurocult-A (StemCell #05751) supplemented with GlutaMAX (Gibco #32050-061), sodium pyruvate (Gibco #11360-070), N2 (Gibco #17502-048), and B27-A (Gibco #12587-010), and fed twice weekly with 20ng/mL EGF (Peprotech #AF-100-15) and 20 ng/mL FGF (Peprotech #100-18C). SF8249 (male, passages 8–14) and SF9030 (male, passage 11–14) were cultured on laminin-coated plates (Gibco #23017-015) in Neurocult-A (StemCell #05751) supplemented with GlutaMAX (Gibco #32050-061), sodium pyruvate (Gibco #11360-070), N2 (Gibco #17502-048), and B27-A (Gibco #12587-010), and fed twice weekly with 20ng/mL EGF (Peprotech #AF-100-15) and 20 ng/mL FGF (Peprotech #100-18C) as previously described (Fouse et al., 2014). All lines were mycoplasma tested and STR validated upon acquisition as previously described (Mancini et al., 2018; Amen et al., 2021).

### METHOD DETAILS

#### Quantitative PCR

RNA was collected with Cells-to-CT kit (ThermoFisher Scientific, #4402955) and reverse transcribed per kit conditions for high throughput (96 well) applications. For low throughput applications, RNA was isolated via Zymo Research Quick-RNA Microprep (#R1051) and cDNA was prepared from 333 ng RNA with iScript cDNA Synthesis Kit (Bio-Rad #1708891). qPCR was performed with PowerSybr Green PCR MasterMix (ThermoFisher Scientific #4368577) on an Applied Biosystems QuantStudio 5 Real-Time PCR System. Relative expression levels were calculated by 2^−ΔΔCt^ analysis and normalized within each replicate to control condition.

#### Western blotting

Protein was harvested using M-PER Mammalian Protein Extraction Reagent (ThermoFisher #78501) supplemented with Turbonuclease (Sigma-Aldrich #T4330) and Halt Protease and Phosphatase Inhibitor (ThermoFisher #78446). Protein was quantified with a BCA assay (ThermoFisher #23225) 20-40 μg of protein was loaded into NuPAGE Tris-Acetate 3–8% gels (ThermoFisher #EA0375) and separated via electrophoresis on XCell SureLock Mini-Cell before transfer to Immobilon PVDF Membrane (Millipore #IPVH00010) in XCell II Blot Module. Blocking, primary, and secondary antibody incubation was performed in 5% BSA in 0.1% Tween 20 tris-buffered saline (Sigma-Aldrich #A9647). Membranes were incubated for 5 min in ECL Western Blotting Substrate (ThermoFisher #32109) before chemilumiescent detection with x-ray film. Background-subtracted signal was quantified via densitometry using ImageJ software.

#### Chromatin immunoprecipitation

ChIP was performed using the ActiveMotif ChIP-IT High Sensitivity kit (ActiveMotif #53040). Cells were grown in two 15 cm plates per condition to 80% confluency and fixed using 4% paraformaldehyde for 15 min. Chromatin was sheared in 30 s on/90 s off intervals for 70 min for a total of 17.5 min of shearing using a Bioruptor sonication device to a size of 200-1500 bp (Diagenode). ChIP was performed for GABPA (Millipore Sigma: ABE1047) antibody or IgG isotype control (Cell Signaling Technologies: 2729) using 30 μg of chromatin and 4ug of antibody per immunoprecipitation reaction. Reactions were incubated at 4°C overnight before proceeding with DNA purification per kit instructions. qPCR was performed using ssoAdvanced Universal SYBR Green Supermix (Biorad #1725270) supplemented with 1M of Resolution Solution from Roche GC-Rich PCR System (Roche #12140306001) with the following cycling protocol: 1. 95°C for 5 min, 2. 95°C for 15 s, 3. 72°C for 60 s, 4. Repeat steps 2–3 39 times. qPCR was performed on an Applied Biosystems QuantStudio 5 Real-Time PCR System. Primers can be located in Supplementary Excel Table.

#### CRISPR sgRNA design and editing

sgRNAs were designed using GuideScan 1.0 (Perez et al., 2017). To generate full gene knockouts, pairs of guides were chosen flanking the coding region. Guides were subcloned into pSpCas9(BB)-2A-Puro (PX459) V2.0 (Addgene #62988). 250 ng of each guide were transfected in tandem with 1.5μL XtremeGene-HP DNA Transfection Reagent. Cells were selected in 1 μg/mL puromycin for 48 h 6 pairs of guides (three 5′ sgRNA and two 3′ sgRNA) were screened by PCR after bulk transfection for editing efficiency and one pair was selected for clone generation. Cells were plated in 96 wells at a concentration of 0.5 cells/100 μL for clone generation.

Clones were screened by PCR with Q5 High-Fidelity DNA Polymerase (NEB #M0491). Separate PCRs were performed for WT and deleted alleles using the same forward primer. For WT alleles, one primer was nested within the gene. For deleted alleles, the primers flanked the entire gene. Clones were then screened by mRNA and protein, compared to parental cells, for loss of expression.

#### TCGA and MSK-IMPACT data access

Processed TCGA RNA-Seq, RPPA, and GISTIC copy number data as well as processed hybridization capture-based mutation and copy number information from MSK-IMPACT was accessed via cBioPortal (Cerami et al., 2012; Gao et al., 2013).

#### Spatially mapped sample collection and data processing

During surgical resection of the tumor, spatially mapped sample coordinates were acquired by a neurosurgeon using Brainlab Cranial Navigation software, which records LPS (left, posterior, superior) sample coordinates on a preoperative MRI. T2/FLAIR MRI was used to preoperatively define the tumor lesion. The samples were collected by a surgeon using a pituitary rongeur, and sampled to maximize total tumor geography coverage. A member of the UCSF Brain Tumor Center Biorepository was present at surgery to ensure the samples were optimally preserved for downstream sequencing.

Brain extraction from the T2-weighted FLAIR image stack was performed using FSL’s Brain Extraction Tool. Brainlab or Slicer was used to draw a tumor ROI for each patient from the T2-weighted hyperintense region.

Sample coordinates were extracted from the screenshots from the surgery with Google’s Cloud Vision API. The numbers were then manually confirmed against coordinates from the corresponding DICOM image from the operating room. Five mm spherical ROIs were assigned to each sample to properly visualize for analysis and publication.

Samples were flash frozen in liquid nitrogen before RNA/DNA collection. Exome capture was performed using the Nimblegen SeqCap EZ Exome v3 (Roche) (P413 and P454) and using the XGen Exome Research Panel v2 (P498 and P500) according to manufacturer’s protocol. Sequencing for both exome and RNA-seq was performed on either a HiSeq2000, HiSeq4000, or NovaSeq. Tumor cell purity was estimated using FACETS from whole exome sequencing data (Shen and Seshan 2016). RNA-seq data from multi-sample human GBM was performed as previously described. Briefly, genomic DNA and RNA were extracted from the same tissue sample using an AllPrep DNA/RNA/miRNA Universal Kit (Qiagen #80234). Libraries were prepared using the Kapa Stranded mRNA-Seq Kit (Kapa Biosystems #KR0960-v2.14). Alignment to hg19 was performed with TopHat v2.0.12 using a GENCODE V19 transcriptome-guided alignment and featureCounts v1.4.6 was used to calculate reads per gene.

#### Plasmids

PRKAB1 (#1:TRCN0000004770 and #2:TRCN0000004771), EGFR (#1:TRCN00000010329 and #2 TRCN0000039634) and non-targeting control shRNA (SHC016) were purchased from Sigma MISSION shRNA. EGFRvIII CDS was synthesized by Twist Bioscience and subcloned into pCW57.1-MCS1-P2A-MCS2 Neo (Addgene #89180) for doxycycline-inducible expression. pDONR223-PRKAA2 (Addgene #23671) was cloned into pLX301 (Addgene #25895) with Gateway LR Clonase II Enzyme Mix (ThermoFisher #11791020). PRKAA2-D157A was generated using QuikChange Lightning Site Directed Mutagenesis Kit (Agilent #210518). *TERT* CDS was subcloned into N174-UBC-Int1-MCS-IRES-Neo.

#### Lentiviral production

Lentivirus was produced in 293T using second generation packaging plasmids, envelope plasmid pMD2.G (Addgene #12259) and packaging plasmid psPAX2 (Addgene #12260). Particles were prepared in 6 well plates using transfection reactions of 1 μg transfer vector, 0.75 μg packaging vector and 0.25 μg envelope vector in serum free media with addition of 0.6μL XtremeGene-HP DNA Transfection Reagent (Roche # 6366546001) for a 3:1 molar ratio. Media was changed 12–16 h after transfection and virus was harvested 48 h later. Viral supernatant was filtered through 0.2 μm polyethersulfone membrane filters (ThermoFisher #720–1320). Selection of stable cell lines was performed for 24 h after viral transduction under 0.5-1 μg/mL puromycin (ThermoFisher #A1113803) or 500ng/mL G418 (ThermoFisher #10131035) as applicable.

#### Luciferase reporter assays

WT (Addgene #84924), C228T (Addgene #84926), and C250T (Addgene #84925) *TERTp* luciferase reporter constructs were used as previously described with Dual-Luciferase Reporter Assay System (Promega #E1910)^15^. Briefly, 3000 cells/well were plated into 96 well white plates and transfected 24 h later with 90 ng *TERT* reporter vector, 9 ng pGL4.74 and 0.3uL XtremeGene-HP DNA Transfection Reagent and read 48 h later. Each condition was plated into six wells for each of three biological replicates.

#### siRNA knockdown

siGENOME SMARTpools were purchased from Dharmacon–non-targeting (D-00206-13-20), GABPA(M-011662-01), and PRKAB1 (M-007675-00-0005). Cells were transfected with a molar ratio of 5:1 siRNA to Dharmafect 1 and harvested 72 h post-transfection.

#### Telomerase repeat amplification protocol

TRAP assays were performed as previously described (Mender and Shay 2015), at a concentration of 2,500 cells/uL. 1 uL lysate was used for each amplification, with 1uL NP-40 lysis buffer serving as a negative control. Oligonucleotides were synthesized by Integrated DNA Technologies. Cy5 signal was visualized on a BioRad ChemiDoc Imager and background-subtracted signal was quantified via densitometry using ImageJ software.

#### Telomere restriction fragmentation (TRF)

Telomere restriction fragmentation was performed using the Telo TAGGG Telomere Length Assay Kit (Roche 12209136001). 1–1.5 μg restricted genomic DNA was separated in 0.8% Ultra High MW agarose in TAE for 2 to 4 h. Gels were incubated with 0.5% HCl and denatured and neutralized as per kit conditions. After overnight transfer to nylon membrane with 20× SSC, the DNA was crosslinked using UV before proceeding with kit protocol.

#### Flow cytometry

Cells were dual stained with FxCycle PI/RNAse staining solution (ThermoScientific #F10797) for 15 min and FITC-conjugated Ki67 (ThermoFisher #11-5699-82) for 45 min. Cells were sorted and analyzed on a FACS Aria II cell sorter using a 488 nm excitation, 530 nm bandpass filter and 561 nm excitation, 620 nm bandpass filter. A minimum of 10,000 cells per condition were analyzed and 100,000 cells were sorted directly into RNA lysis buffer.

### QUANTIFICATION AND STATISTICAL ANALYSIS

For testing of significance, two-tailed student’s t-tests were used for RT-qPCR, western blotting, and luciferase assays for three separate biological replicates. For TCGA analyses, Wilcoxon rank-sum tests were used. p values < .05 were considered significant. For association between EGFR amplification and *TERT*p mutations, a two-tailed Fisher exact probability test was utilized. For comparisons between three or more conditions, a one-way ANOVA was performed to test for global significance. If significance was reached, individual two-tailed student’s t-tests were used to test individual comparisons. Statistical details for each test can be found in the figure legends. Significance tests were performed using GraphPad Prism 9 software.

## Supplementary Material

1

2

3

4

## Figures and Tables

**Table:** KEY RESOURCES TABLE

REAGENT or RESOURCE	SOURCE	IDENTIFIER
Antibodies		
p-EGFR (Y1068)	Cell Signaling Technology	Cat# 2234, RRID:AB_331701
EGFR	Cell Signaling Technology	Cat# 4267, RRID:AB_2246311
GABPA	Millipore Sigma	Cat# ABE1047, RRID:AB_2921697
GABPB1	Proteintech	Cat# 12597-1-AP, RRID:AB_10951115
GAPDH	Millipore Sigma	Cat# CB1001, RRID:AB_2107426
p-AMPKA (T172)	Cell Signaling Technology	Cat# 2535, RRID:AB_331250
AMPKA1/2	Cell Signaling Technology	Cat# 2532, RRID:AB_330331
AMPKB1/2	Cell Signaling Technology	Cat# 4150, RRID:AB_10828832
Anti-rabbit IgG, HRP-linked	Cell Signaling Technology	Cat# 7074, RRID:AB_2099233
Anti-mouse IgG, HRP-linked	Cell Signaling Technology	Cat# 7076, RRID:AB_330924
Rabbit IgG	Cell Signaling Technology	Cat#2729, RRID:AB_1031062
Ki67 Monoclonal Antibody (20Raj1), FITC, eBioscience	ThermoFisher	Cat# 14-5699-82, RRID:AB_2016711)
Biological samples		
Patient 413 spatially mapped glioblastoma samples	This paper	N/A
Patient 454 spatially mapped glioblastoma samples	This paper	N/A
Patient 498 spatially mapped glioblastoma samples	This paper	N/A
Patient 500 spatially mapped glioblastoma samples	This paper	N/A
Chemicals, peptides, and recombinant proteins		
XtremeGene-HP DNA Transfection Reagent	Roche	Cat #6366546001
ssoAdvanced Universal SYBR Green Supermix	Bio-Rad	Cat #1725270
Resolution Solution from GC-Rich PCR System	Roche	Cat# 19024024
Dharmafect 1	Horizon	Cat #T-2001-02
Q5 High Fidelity DNA Polymerase	New England Biolabs	Cat #M0491
FxCycle PI/RNAse Staining Solution	ThermoFisher	Cat ##F10797
Critical commercial assays		
Power SYBR Cells to CT	ThermoFisher	Cat #4402955
ChIP-IT High Sensitivity	Active Motif	Cat #53040
Dual Luciferase Reporter Assay System	Promega	Cat #E1910
TeloTAGGG Telomere Length Assay Kit	Roche	Cat #12209136001
Quick-RNA Microprep	Zymo Research	Cat #R1051
iScript cDNA Synthesis Kit	Bio-Rad	Cat #1708891
BCA Assay	ThermoFisher	Cat #23225
AllPrep DNA/RNA/miRNA Universal Kit	Qiagen	Cat #80234
Stranded mRNA-Seq Kit	Kapa Biosystems	Cat #KR0960-v2.14
QuikChange Lightning Site Directed Mutagenesis Kit	Agilent	Cat #210518
Gateway LR Clonase II Enzyme Mix	ThermoFisher	Cat# 11791020
Deposited data		
RNA-seq data, spatially mapped samples	European Genome-Phenome Archive	EGA00001003710
Exome data, spatially mapped samples	European Genome-Phenome Archive	EGA00001003710
Experimental models: Cell lines		
SF7996	Fouse et al. (2014)	N/A
LN229	ATCC	Cat# CRL-2611; RRID: CVCL_0393
NHAPC5	Ohba et al. (2016)	N/A
SF8249	Fouse et al. (2014)	N/A
SF9030	Fouse et al. (2014)	N/A
LN18	ATCC	Cat# CRL-2610; RRID: CVCL_0392
U251	ECACC	Cat# 09063001, RRID:CVCL_0021
GBM6	Sarkaria et al. (2006)	N/A
Oligonucleotides		
siRNA non-targeting pool	Dharmacon	D-00206-13-20
siGABPA pool	Dharmacon	M-011662-01
siPRKAB1 pool	Dharmacon	M-007675-00-0005
shPRKAB1_1	Sigma Aldrich	Cat # TRCN0000004770
shPRKAB1_2	Sigma Aldrich	Cat # TRCN0000004771
shEGFR_1	Sigma Aldrich	Cat # TRCN0000010329
shEGFR_2	Sigma Aldrich	Cat # TRCN0000039634
shScr	Sigma Aldrich	SHC016
pSpCas9(BB)-2A-Puro (PX459) V2.0	Addgene	RRID:Addgene_62988
pCW57.1-MCS1-P2A-MCS2 Neo	Addgene	RRID:Addgene_89180
pDONR223-PRKAA2	Addgene	RRID:Addgene_23671
pLX301	Addgene	RRID:Addgene_25895
pMD2.G	Addgene	RRID:Addgene_12259
psPAX2	Addgene	RRID:Addgene_12260
pGL4.0-TERT WT	Addgene	RRID:Addgene_84924
pGL4.0-TERT G228A	Addgene	RRID:Addgene_84926
pGL4.0-TERT G250A	Addgene	RRID:Addgene_84925
Prism	Graphpad	https://www.graphpad.com/how-to-buy/
ImageJ	ImageJ	https://imagej.nih.gov/ij/download.html
FlowJo	FlowJo LLC	https://www.flowjo.com/solutions/flowjo/downloads

## Highlights

- EGFR signaling leads to activation of the mutant *TERT* promoter in GBM
- Cell cycling is functionally linked with regulation of the mutant *TERT* promoter
- EGFR upregulates the mutant TERT promoter via AMPK and GABP, maintaining telomere length
- Clinical inhibitors of EGFR reduce GABP binding and TERT expression
