# Supplementary material for: GABP couples oncogene signaling to telomere regulation in TERT promoter mutant cancer
Source: Cell Rep. Author manuscript; Available in PMC 2022 Oct 5. (PMC9534059; doi:10.1016/j.celrep.2022.111344)
Supplement: 1 [file NIHMS1837776-supplement-1.pdf]

Cell Reports, Volume *40*

Supplemental information

GABP couples oncogene signaling to telomere regulation in TERT promoter mutant cancer

Andrew M. McKinney, Radhika Mathur, Nicholas O. Stevers, Annette M. Molinaro, Susan M. Chang, Joanna J. Phillips, and Joseph F. Costello

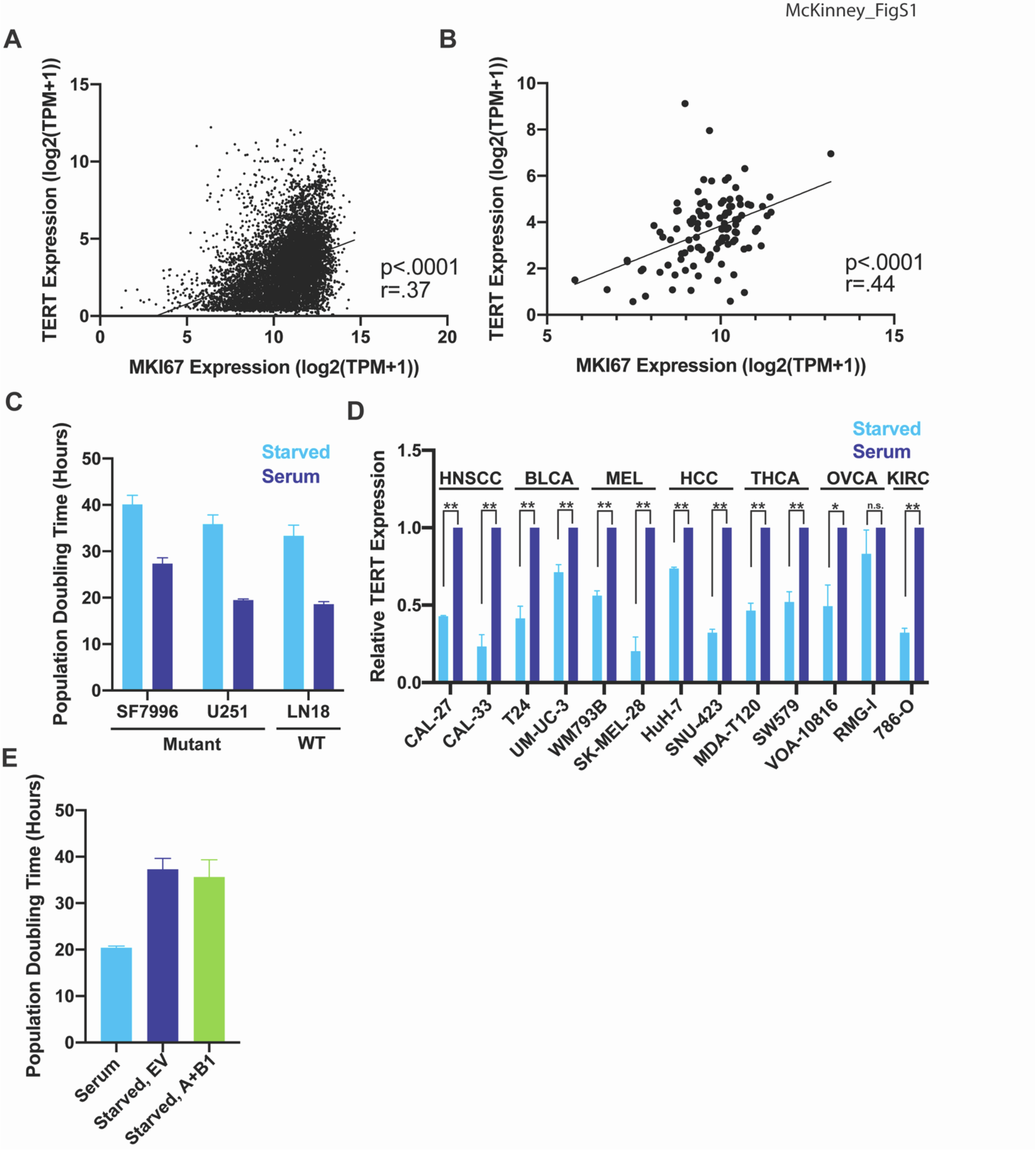

Supplementary Figure 1. ***TERT*** **expression level is associated with proliferation in cancer.** *(A-B)* Correlation of log2 RNA-Seq of pan-cancer *(A)* and *IDH1*-WT GBM *(B)* tumors from TCGA of MKI67 and TERT. r, Pearson correlation coefficient, p<0.0001. *(C)* Population doubling in hours upon serum starvation in *TERT*p-WT and *TERT*p-mut cells for 24 hours. *(D) TERT* expression in serum starved *TERT*p-mut cells and after 24 hours serum induction in head and neck squamous cell carcinoma (HNSCC), urinary bladder cancer (BLCA), melanoma (MEL), hepatocellular carcinoma (HCC), thyroid cancer (THCA), ovarian cancer (OVCA), and renal cell carcinoma (KIRC) cell lines. n=3 biological replicates, Student's t-tests, two-tailed. *P<0.05, **P<.005, data represent mean +/-

SEM, n.s., non-significant. *(E)* Population doubling in hours following 24 hours of serum starvation in U251 cells expressing ectopic *GABPA* and *GABPB1* or empty vector control. Related to Figure 1.

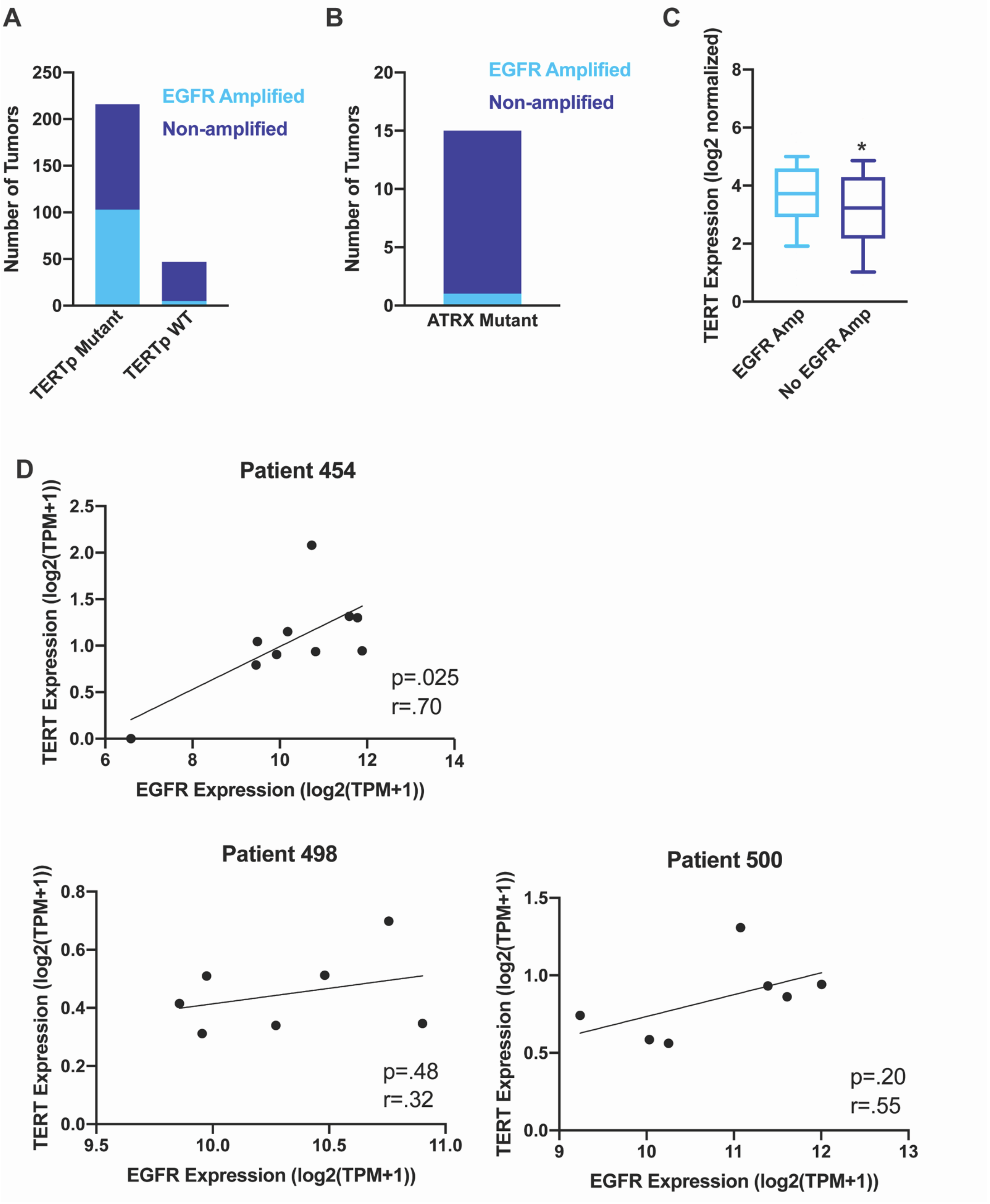

Supplementary Figure 2. *TERT* expression correlates with EGFR amplification and EGFR expression in GBM.
(A) Number of *IDH1*-WT GBM harboring EGFR amplification in *TERT*p mutant or *TERT*p-WT tumors in MSK-IMPACT data (216 samples) Fisher exact probability test, two tailed, p<0.0001**.** *(B)* Number of

IDH1-WT GBM tumors harboring EGFR amplification in ATRX mutant cancers in MSK-IMPACT data (15 samples). *(C) TERT* mRNA expression in EGFR amplified or non-amplified *IDH1*-WT GBM tumors (TCGA data). Tumors with available RNA-Seq data and tumor purity greater than 60% were stratified into 61 EGFR amplified and 46 non-amplified cases. Whiskers represent 5th and 95th percentile values. Wilcoxon rank-sum test, two-tailed. *P<0.05, **P<.005. *(D)* Correlation of log2-normalized relative EGFR and *TERT* expression within 7-10 intratumoral samples (tumor purity greater than 60%) from each of three EGFR amplified GBM. r, Pearson correlation coefficient. Related to Figure 2.

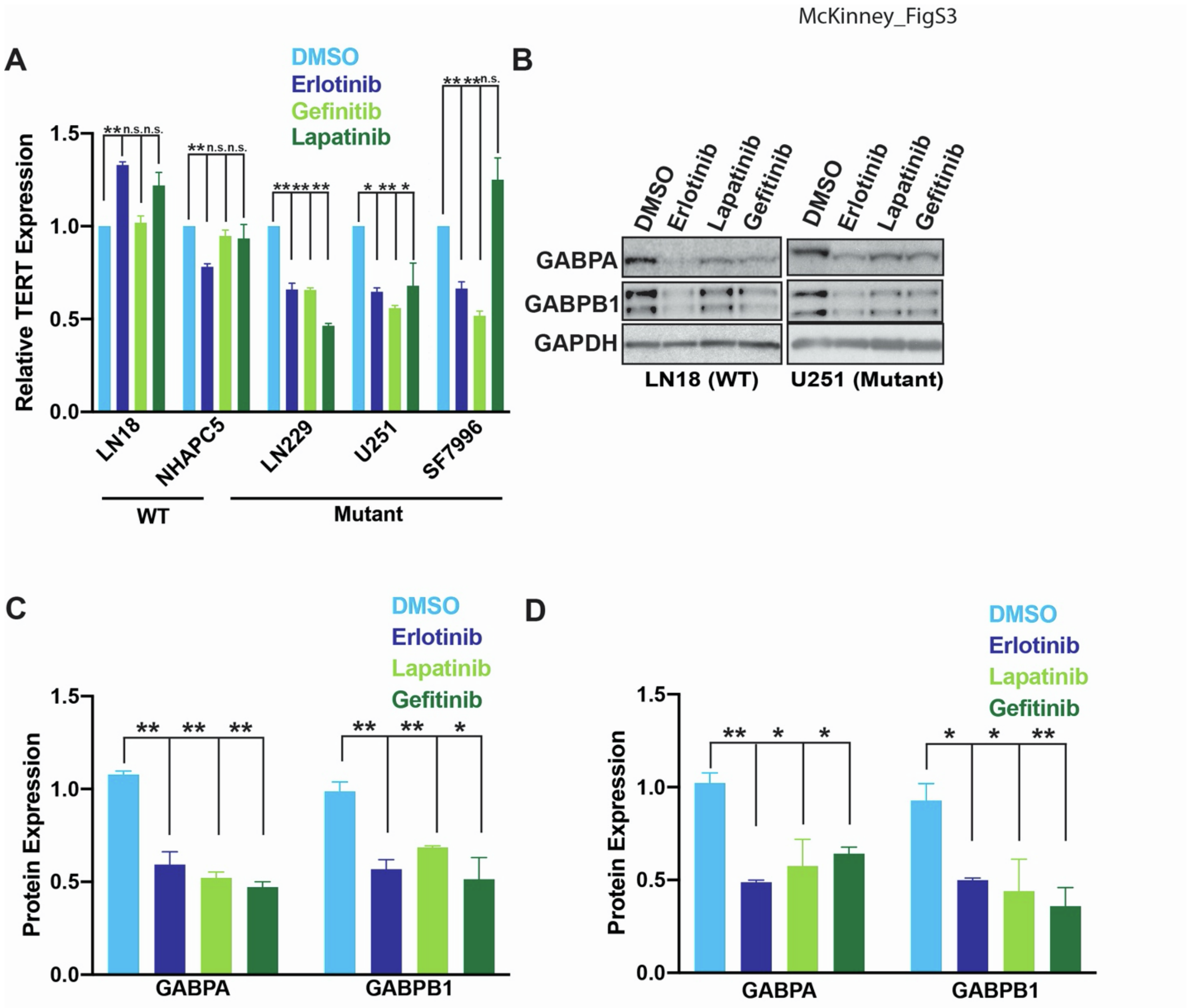

Supplementary Figure 3. Pharmacological inhibition of EGFR downregulates the GABP-TERT axis.

(A) TERT expression after pharmacological EGFR inhibition (5uM for all three drugs) measured by RT-qPCR in *TERT*p-WT and *TERT*p-mut cell lines, normalized relative to DMSO in each cell line, n=3. *(B)* Representative immunoblots of GABPA and GABPB1 upon pharmacological EGFR inhibition compared to DMSO (leftmost lane) in *TERT*p-WT and *TERT*p-mut cell lines. *(C-D)* Quantification of TERTp-WT *(C)* and TERTp-mut *(D)* immunoblots of GABP subunits from panel B, n=2 biological replicates. *(A-D)* Student's t-tests, two-tailed. *P<0.05, **P<.005, data represent mean +/- SEM, n.s., non-significant. Related to Figures 2 and 3.

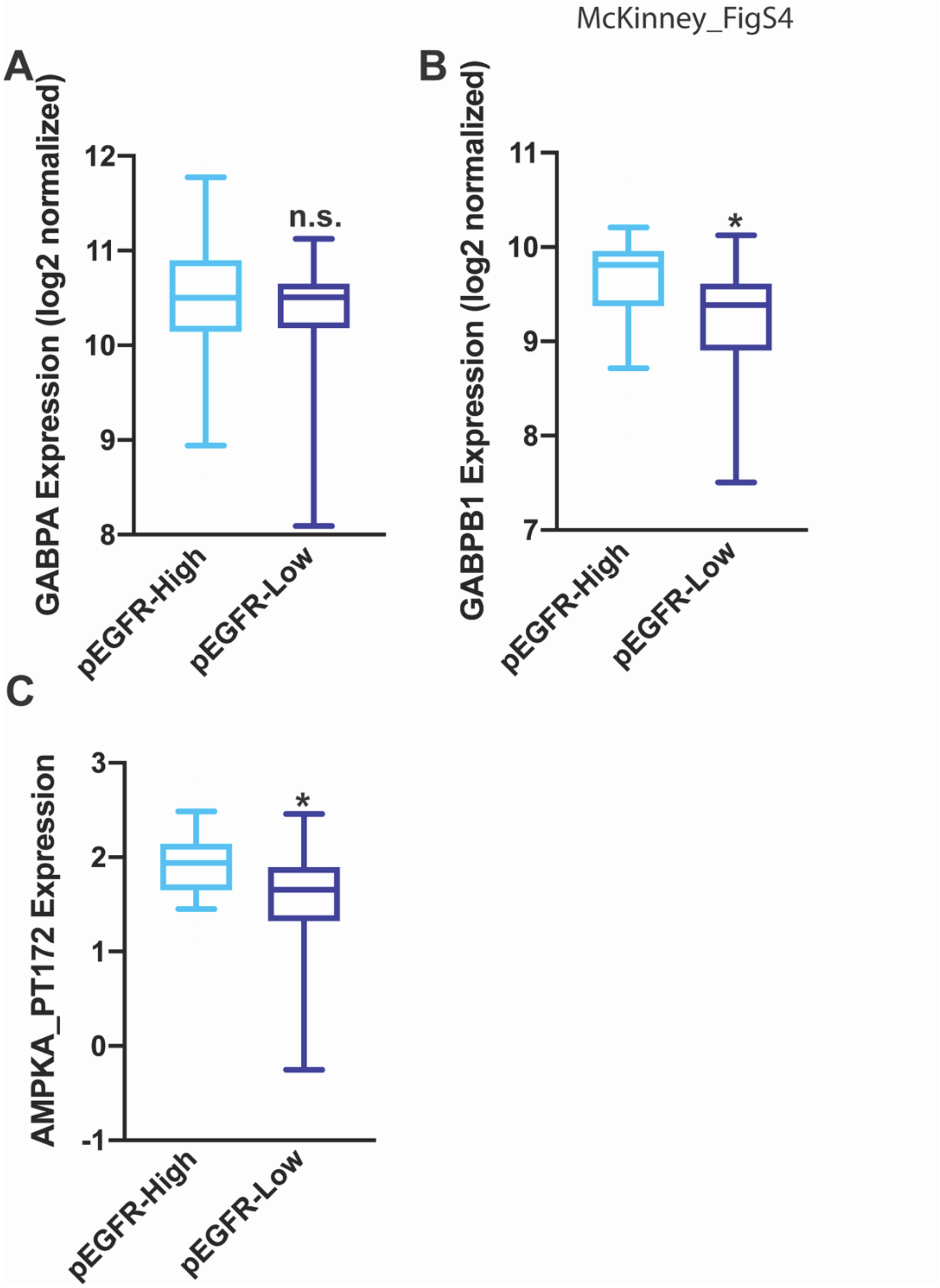

Supplementary Figure 4. GABP and p-AMPK are elevated in EGFR-high GBM.
(A) GABPA mRNA expression in *IDH1*-WT GBM. Tumors profiled by TCGA with available RNA-Seq and RPPA data were stratified into 27 EGFR-high, 27 EGFR-low. *(B) GABPB1* mRNA expression in *IDH1*-WT GBM tumors. Tumors profiled by TCGA with available RNA-Seq and RPPA data were stratified into 27 EGFR-high, 27 EGFR-low. *(C)* Expression of endogenous AMPKA1_PT172 in EGFR amplified or non-amplified *IDH1*-WT GBM. Tumors profiled by TCGA with available RNA-Seq and RPPA data were stratified into 27 EGFR-high, 27 EGFR-low cases. (A-C) Samples with less than 60% tumor purity were excluded. Whiskers represent 5th and 95th percentile values. Wilcoxon rank-sum test, two-tailed. *P<0.05, **P<.005, n.s., non-significant. Related to Figures 3 and 5.

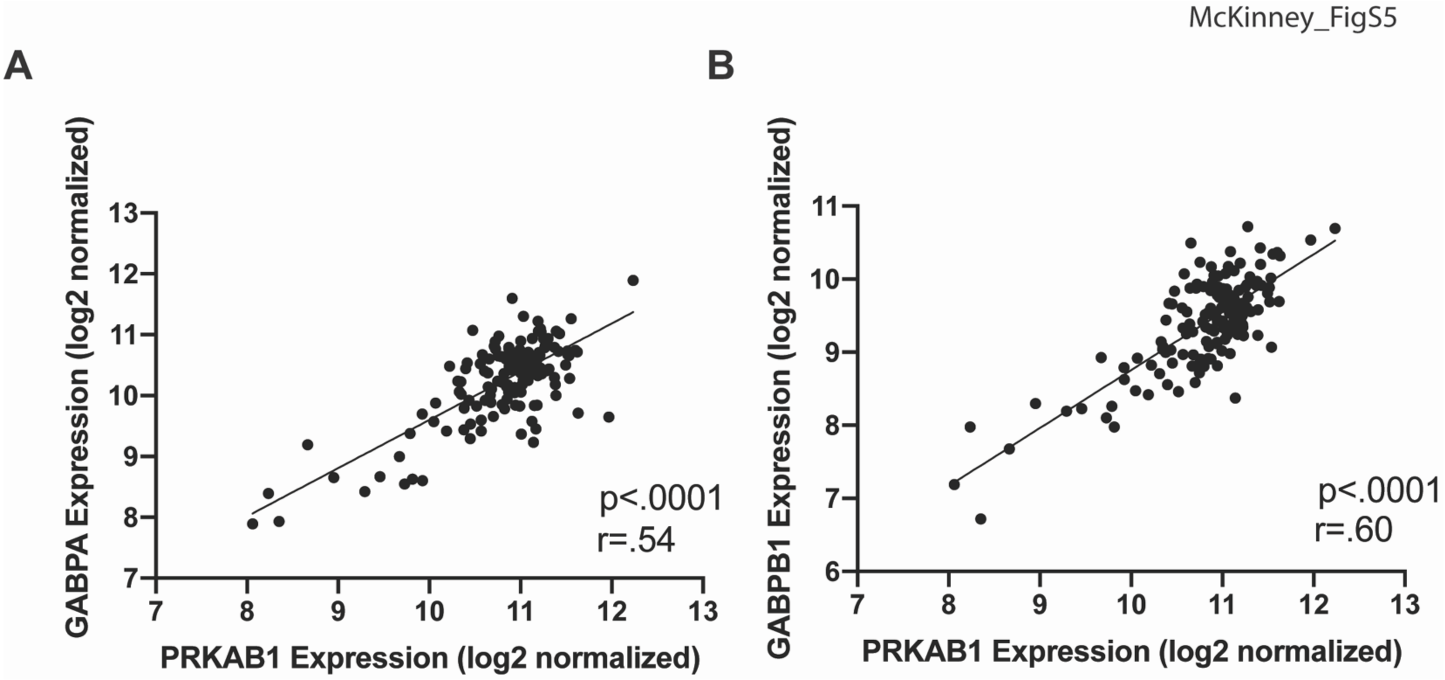

Supplementary Figure 5. PRKAB1 correlates with GABP subunit expression in GBM.
(A-B) Correlation of PRKAB1 with *GABPA* expression *(A)* and *GABPB1* expression *(B)* in GBM profiled by TCGA. r, Pearson coefficient. Related to Figure 5.

McKinney_FigS6

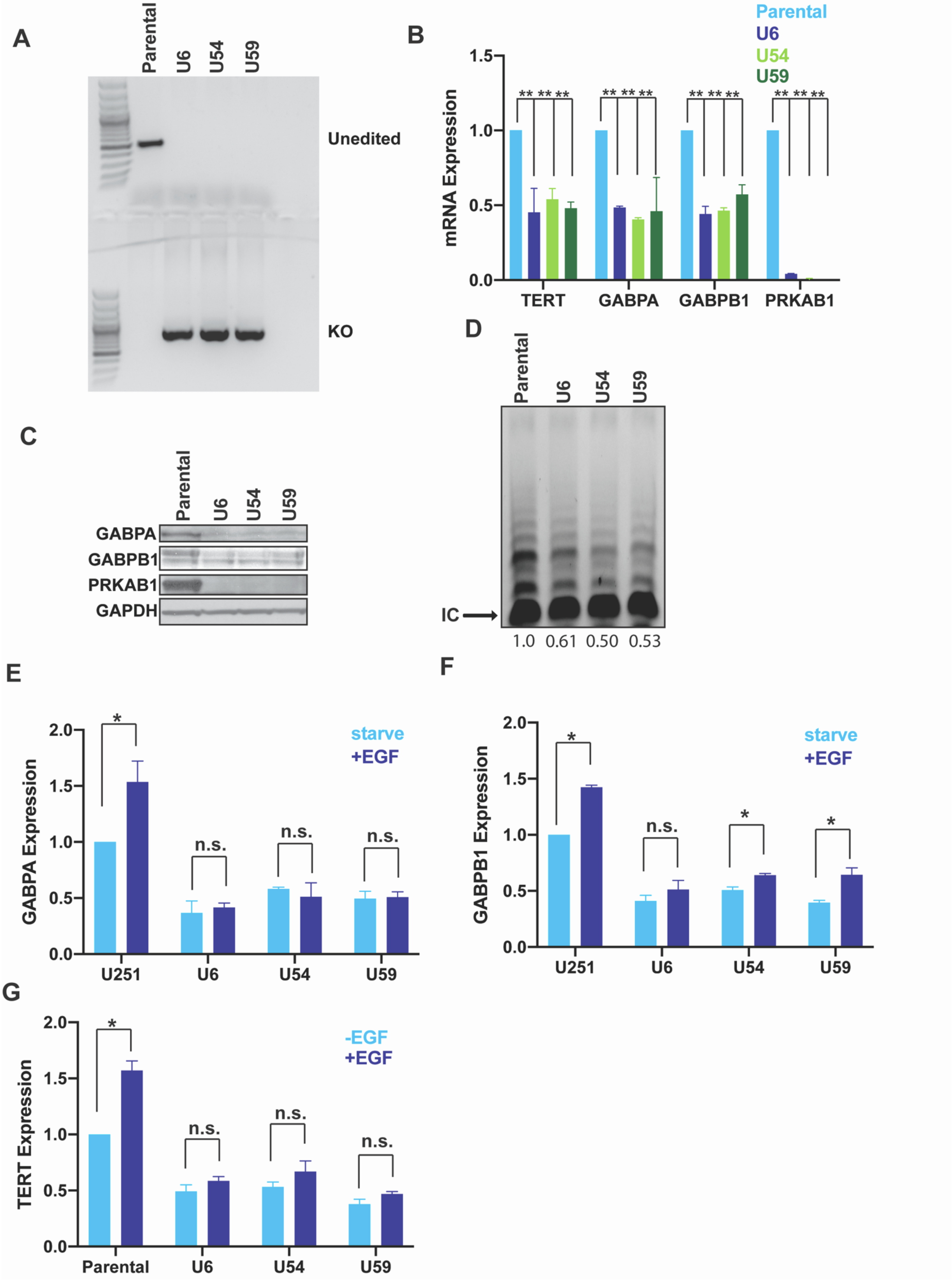

Supplementary Figure 6. CRISPR-mediated full gene knockout of PRKAB1 attenuates EGFR induction of GABP and TERT expression.
(A) Genotyping analysis of PRKAB1 full gene knockout clones (U6, U54, and U59) compared to parental U251 cells. *(B)* mRNA levels of *TERT, GABPA, GABPB1* and *PRKAB1* in U251 *PRKAB1* full gene knockout clones measured by RT-qPCR, relative to parental, unedited U251 cells, n=3 technical replicates. *(C)* Immunoblots of GABPA, GABPB1, and PRKAB1 in U251 *PRKAB1* total knockout clones. *(D)* Telomerase activity of U251 *PRKAB1* total knockout clones. (*E-G) GABPA, GABPB1,* and *TERT* expression in U251 *PRKAB1* full gene knockout clones measured by RT-qPCR, relative to parental, unedited U251 cells after 12 hours of induction with EGF, n=3 biological replicates *(B, E-G)* Student's t-tests, two-tailed. *P<0.05, **P<.005, data represent mean +/- SEM, n.s., non-significant. Related to Figures 5 and 6.

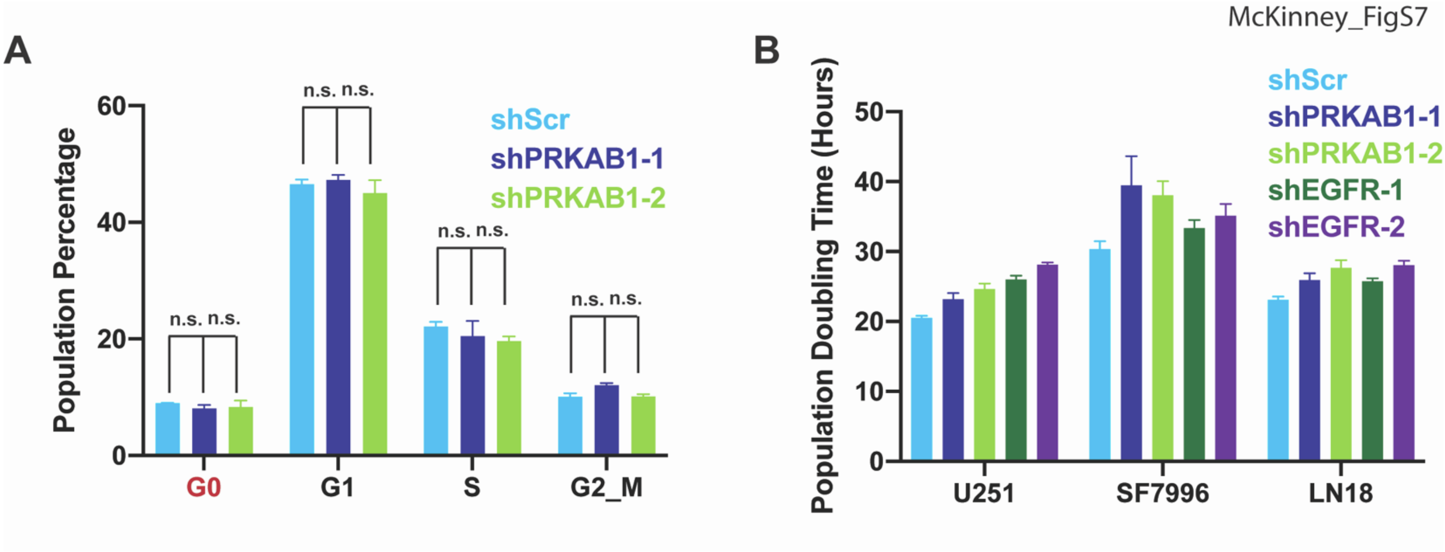

Supplementary Figure 7. Effects of EGFR and PRKAB1 knockdown on cell cycle and proliferation rate

(A) Percentage of cells in phases of the cell cycle (G0, G1, S, G2_M) after shRNA targeting of PRKAB1, as measured by flow cytometry of PI and KI67, n=3 biological replicates. (B) Population doubling in hours upon shRNAs knockdown of *PRKAB1* or *EGFR*, n=3 biological replicates. Related to Figure 7.
